# Genomic features of the polyphagous cotton leafworm *Spodoptera littoralis*

**DOI:** 10.1186/s12864-022-08582-w

**Published:** 2022-05-07

**Authors:** Chao Wu, Lei Zhang, Bo Liu, Bojia Gao, Cong Huang, Ji Zhang, Minghui Jin, Hanyue Wang, Yan Peng, Annabel Rice, Esmat Hegazi, Kenneth Wilson, Pengjun Xu, Yutao Xiao

**Affiliations:** 1 Lancaster Environment Centre, Lancaster University, Lancaster, UK; 2 Shenzhen Branch, Guangdong Laboratory of Lingnan Modern Agriculture; Genome Analysis Laboratory of the Ministry of Agriculture and Rural Affairs; Agricultural Genomics Institute at Shenzhen, Chinese Academy of Agricultural Sciences, Shenzhen, China; 3 Department of Entomology, Faculty of Agriculture Alexandria University, Alexandria, 22542 Egypt; 4 Tobacco Research Institute, Chinese Academy of Agricultural Sciences, Qingdao, China

**Keywords:** *Spodoptera littoralis*, Polyphagy, Comparative genomics, Genetic features

## Abstract

**Background:**

The cotton leafworm, *Spodoptera littoralis*, is a highly polyphagous pest of many cultivated plants and crops in Africa and Europe. The genome of this pest will help us to further understand the molecular mechanisms of polyphagy.

**Results:**

Herein, the high-quality genome of *S. littoralis* was obtained by Pacific Bioscience (PacBio) sequencing. The assembled genome size of *S. littoralis* is 436.55 Mb with a scaffold N50 of 6.09 Mb, consisting of 17,207 annotated protein-coding genes. Phylogenetic analysis shows that *S. littoralis* and its sibling species *S. litura* diverged about 5.44 million years ago. Expanded gene families were mainly involved in metabolic detoxification and tolerance to toxic xenobiotics based on GO (Gene Ontology) and KEGG (Kyoto Encyclopedia of Genes and Genomes) pathway analysis. Comparative genomics analysis showed that gene families involved in detoxification and chemosensation were significantly expanded in *S. littoralis*, representing genetic characteristics related to polyphagy and an extensive host range.

**Conclusions:**

We assembled and annotated the reference genome of *S. littoralis*, and revealed that this pest has the genetic features of strong detoxification capacity, consistent with it being a significant risk to a wide range of host crops. These data resources will provide support for risk assessment and early warning monitoring of major polyphagous agricultural pests.

**Supplementary Information:**

The online version contains supplementary material available at 10.1186/s12864-022-08582-w.

## Background

The genus *Spodoptera* (Lepidoptera: Noctuidae) contains about 31 species spread over six continents, many of which are important agricultural pests worldwide, including African armyworm (*S. exempta*), beet armyworm (*S. exigua*), fall armyworm (*S. frugiperda*) and tobacco cutworm (*S. litura*). Their main hosts include field crops, grasses and vegetables, causing great losses to agricultural production [1–4].

**Fig. 1:**
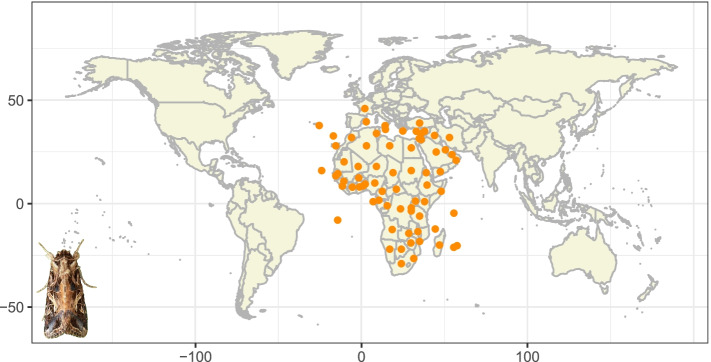
Geographical distribution of *S. littoralis*. This pest is widely distributed in tropical and subtropical regions, specifically in Africa, Mediterranean Europe and the Middle East. The geographic distribution data were obtained from website: https://gd.eppo.int/taxon/SPODLI/distribution, and each dot represents the coordinates of the reported discovery of *S. littoralis*, and the picture of *S. littoralis* is in the lower left corner

Cotton leafworm (*S. littoralis*) is native to Sub-Saharan Africa, and is one of the most destructive agricultural pests in subtropical and tropical areas. At present, this pest is widely distributed throughout Africa, Mediterranean Europe and the Middle East (Fig. 1). The dispersal of *S. littoralis* occurs mainly through trade when eggs or larvae are present in imported ornamental plants or crops [5]. With increasing international trade, this species is at great risk of entering the Americas and Eastern Asia. Now, *S. littoralis* is assigned as an A2 quarantine pest by European and Mediterranean Plant Protection Organization (EPPO) and listed as an exotic organism with high invasive risk in the United States [6, 7].

*S. littoralis* is a highly polyphagous organism that feeds on at least 87 kinds of plants that include a variety of important crops such as tomatoes, peppers, cotton, corn [5, 8]. Damage to cotton plants by this species is most serious and prevalent in North Africa, especially in Egypt [9]. There was a catastrophic outbreak of *S. littoralis* in Spain in 1949, affecting potatoes, lucerne and other vegetable crops [10]. At present, it is also a major pest in areas such as Cyprus, Israel, Malta, and Morocco [11].

The damage capacity of a pest is closely related to their biological characteristics, such as their capacities for host recognition, detoxification, energy storage, etc. Most *Spodoptera* species can feed on various host plants with a wide geographical range. Therefore, they are ideal model organisms for studying environmental and host adaptation.. The genome sequences of *S. exigua*, *S. frugiperda* and *S. litura* have been published in recent years [12–14]. In this study, we sequenced and assembled a high-quality genome of *S. littoralis* collected from Egypt, and the host plant diversity and strong environmental adaptability were revealed through the expansion of detoxification and chemosensation gene families. These resources will provide a genomic basis for assessing the damage risk of *S. littoralis*.

## Results and discussion

### Genome assembly, evaluation

**Fig. 2:**
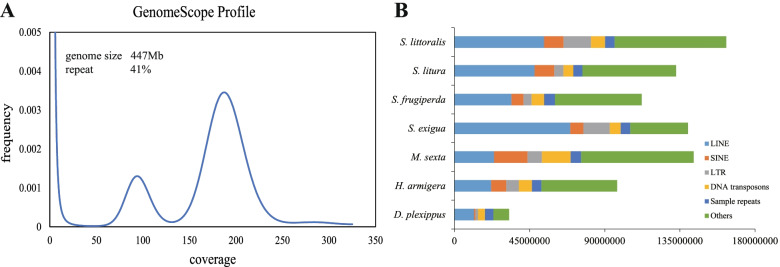
Genomic survey and functional annotation for *S. littoralis*. **a** Genome survey from 17-mer frequency distribution plot estimated for *S. littoralis*. The estimated genome size was 447 Mb, with 41% repeats, is very close to the actual assembled genome size (436.55 Mb) and repeats (37.29%). **b** Comparison of repeated sequences in *S. littoralis* and several other Lepidopteran insects. The total length of repeated sequences and their proportion in the *S. littoralis* genome is higher than that in the other three *Spodoptera* species and more Lepidopteran insects

In this study, a total of ~ 55 gigabases (Gb) (~ 128×) of sequencing data were generated by PacBio long-read technology (Additional file 1: Table S1). Canu version 1.9 software was used to assemble the *S. littoralis* genome. The final genome was assembled into 238 scaffolds with an assembly size of 436.55 Mb, which was comparable to the estimated genome size (447.08 Mb) by the distribution of *k*-mer analysis using Illumina data (Fig. 2a). The genome size of *S. littoralis* was also very similar to that of the three closely related *Spodoptera* species previously reported, *S. litura* (438.32 Mb) [12], *S. frugiperda* (390.38 Mb) [13] and *S. exigua* (446.80 Mb) [14] (Table 1, Additional file 2: Table S2). The contig and scaffold N50 lengths assembled in the current *S. littoralis* genome were 4.35 Mb and 6.07 Mb, respectively. The assembled genome of *S. littoralis* has fewer contigs and scaffolds than that of the other three closely related *Spodoptera* species with assembled genomes (Table 1). The assembled *S. littoralis* genome provides another high-quality reference genome sequence for the study of key characteristics of insects in the genus *Spodoptera*.

**Table 1:** Summary of assembly results of *S. littoralis*

Assembly feature	***S. littoralis***	***S. litura***	***S. frugiperda***	***S. exigua***
Version	this study	GCA_002706865.1	GCA_012979215.2	GCA_011316535.1
Assembly size (Mb)	436.55	438.96	390.40	446.8
Assembly level	Scaffolds	Chromosomes	Chromosomes	Chromosomes
Number of scaffolds	238	3597	666	301
Longest scaffold (Mb)	15.97	4.42	21.91	19.74
Scaffold N50 length (Mb)	6.09	0.91	12.96	14.36
Number of contigs	308	13,637	776	667
Longest contig (Mb)	15.84	0.64	18.55	15.88
Contig N50 length (Mb)	4.35	0.068	5.6	3.47
Number of gene models	17,207	15,317	22,260	17,727
GC content (%)	36.88	36.56	36.4	36.67
BUSCO completeness (%)	98.4	98.5	98.3	97.6
BUSCO duplicated (%)	0.40	0.80	0.90	1.70
BUSCO missing (%)	1.20	1.10	1.50	2.30

To estimate the quality of genome assembly in *S. littoralis*, the Illumina reads were aligned to the assembled scaffolds, resulting in a mapping rate and coverage rate of 99.70 and 98.25% (≥5 reads), respectively. In addition, the Benchmarking Universal Single-Copy Orthologues (BUSCO) analyses showed that the assembled *S. littoralis* genome covered 98.4% complete (including 0.4% complete and duplicated), 0.4% fragmented, and 1.2% missing BUSCO genes (Additional file 3: Fig. S1, Table 1). Compared with the other three *Spodoptera* species, the completeness of this genome assembly was equal to that of *S. frugiperda* assembled by Zhang et al. [13], slightly more complete than *S. frugiperda* and *S. exigua*, and with slightly fewer duplications. The evaluation results indicated a high accuracy and integrity of the reference genome for *S. littoralis*.

### Gene prediction and functional annotation

Based on the evidence of homologous sequence similarity and repeating sequence structure characteristics, a total of 37.29% (162.28 Mb) repetitive elements were identified in the assembled *S. littoralis* genome (Additional file 4: Table S3). Among the known transposable elements (TEs), long interspersed nuclear elements (LINEs) were the most abundant repeat family, accounting for 12.29% of assembled genome, followed by short interspersed nuclear elements (SINE: 2.68%), long terminal repeats (LTR: 3.75%), rolling-circle transposition (RC: 4.35%) and DNA transposons (1.94%) (Fig. 2b, Additional file 4: Table S3). In addition, simple repeats also occupied 1.30% of the assembled genome.

The proportion of repeated sequences in the *S. littoralis* genome (37.29%) is higher than that in the other three *Spodoptera* species: *S. exigua* (31.30%), *S. frugiperda* (28.71%) and *S. litura* (30.24%) [12–14] (Fig. 2b). Compared with other Lepidoptera species with known whole genome sequences, the proportion of repeated elements in *S. littoralis* genomes are generally higher than that in *Helicoverpa armigera* (24.96%), *Danaus plexippus* (13.17%) and *Manduca sexta* (30.48%) [15–17].

To annotate the genome of *S. littoralis*, we performed deep transcriptome sequencing of larvae, pupae, female and male moths, covering three developmental stages. The Evidence Modeler pipeline was used to integrate the evidence of de novo predictions, protein homology, and RNA-seq transcripts. In total, 17,207 protein-coding genes were predicted in the *S. littoralis* genome, with the average coding sequence (CDS) length of 1937 bp and the average exon number of 6.82, and with 14,892 (~ 86.55%) of the protein-coding genes detected in at least one sample of RNA-Seq data. The number of protein-coding genes in *S. littoralis* was similar to that in *S. exigua* (17,727), more than that in *S. litura* (15,317) and fewer than that in *S. frugiperda* (22,260) (Table 1).

Among the predicted genes of *S. littoralis*, a total of 16,182 genes (94.04% of all genes) were annotated in the four functional databases, with 15,992, 15,814, 15,631 and 11,545 genes being annotated by the GenBank NR protein database, TREMBL protein database, EggNOG protein database, and KEGG protein database, respectively (Additional file 5: Fig. S2, Additional file 6: Table S4). In addition, a total of 509 non-coding RNAs (ncRNAs) were identified in the *S. littoralis* genome, including 83 ribosomal RNAs (rRNAs), one small cytoplasmic RNA (scRNA), 157 small nuclear RNAs (snRNAs), and 268 transfer RNAs (tRNAs).

### Orthologous genes and comparative genomic analysis

To further understand the evolutionary perspective of *Spodoptera* species including *S. littoralis*, twelve species of Lepidoptera were selected for whole genome-wide phylogenomic analyses, including three butterflies (*D. plexippus*, *Heliconius melpomene*, *Papilio xuthus*), and nine moths (Noctuidae: *S. exigua*, *S. frugiperda*, *S. littoralis*, *S. litura*, *H. armigera*, *Trichoplusia ni*; Bombycidae: *B. mori*, *Manduca sexta*; Plutellidae: *Plutella xylostella*), with *Drosophila melanogaster* (Diptera, Drosophilidae) being selected as the outgroup.

**Fig. 3:**
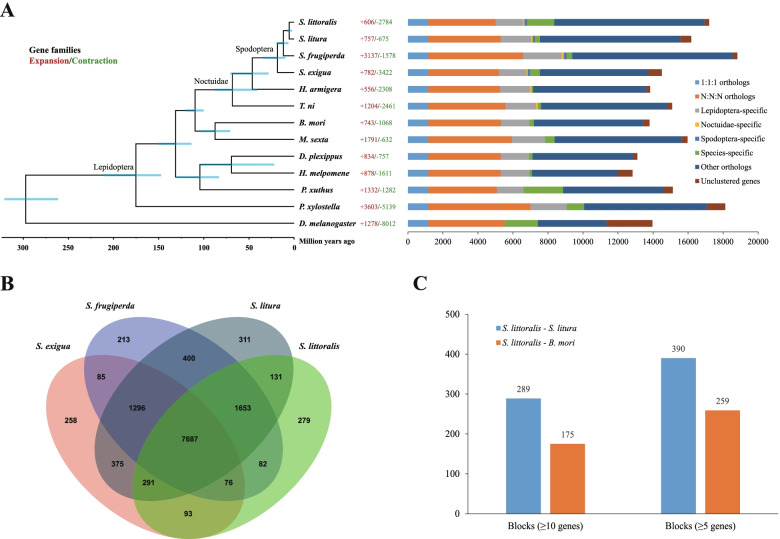
Genome evolution of *S. littoralis*. **a** Phylogenetic species tree, divergence time and expansion and contraction of gene families in thirteen species. Divergence times calculated by MCMCtree, and the blue bars at the internodes indicate the 95% confidence intervals of the divergence time. Genomic comparison of thirteen sequenced species. 1:1:1 orthologs include the common orthologs with a single-copy in each species, N:N:N orthologs include the common orthologs with variable copy numbers in the different species. **b** The comparison of homologous genes in four species of the same genus *Spodoptera*. the number of species-specific genes in *S. littoralis* was more than that in *S. frugiperda* and *S. exigua*, and less than that in *S. litura*. **c** The number synteny blocks of *S. littoralis* – *S. littura* and *S. littoralis* – *B. mori*. The number of synteny blocks between *S. littoralis* and *S. litura* was more than that between *S. littoralis* and *B. mori*

OrthoFinder was used to analyze the homology of protein-coding genes of the 13 species. A total of 198,013 genes were clustered into 15,202 orthogroups. Of these, 16,941 genes of *S. littoralis* were clustered into 10,292 orthogroups, and among them 1254 orthogroups contained at least two genes. 3842 N:N:N genes, 145 *Spodoptera*-specific genes, and 1544 species-specific genes were identified in *S. littoralis* (Fig. 3a, Additional file 7: Table S5, Additional file 8: Table S6). For the four representative *Spodoptera* species, the number of species-specific genes in *S. littoralis* was 279, which was more than that in *S. frugiperda* (213 genes) and *S. exigua* (258 genes), and less than that in *S. litura* (311 genes) (Fig. 3b).

A phylogenetic tree was constructed for the 13 species using 1170 single-copy orthologous genes. In the Noctuidae family, the four *Spodoptera* species (*S. exigua*, *S. frugiperda*, *S. littoralis* and *S. litura*) grouped together in a clade. *T. ni*, an early divergent species, is located at the base of the phylogenetic tree of Noctuidae (Fig. 3a). Moreover, two calibration points were selected to infer the divergence times of the 13 species. The results showed that the genus *Spodoptera* separated from the family Noctuidae approximately 46.34 million years ago. *S. littoralis* is most closely related to *S. litura*, possibly diverging 5.44 million years ago. *S. littoralis* and *S. frugiperda* may have diverged from their common ancestor approximately 11.84 million years ago. *S. exigua* and *S. frugiperda* may have diverged approximately 18.36 million years ago (Fig. 3a). Genome synteny analysis showed that the number of synteny blocks (≥5 genes) between *S. littoralis* and *S. litura* was 390, which is more than that between *S. littoralis* and *B. mori* (only 259 blocks) (Fig. 3c, Additional file 9: Table S7). To some extent, due to the higher similarity between *S. littoralis* and *S. litura* in genome, the result of synteny analysis again confirms the accuracy of *S. littoralis* genome assembly and gene prediction.

### Rapidly expanded gene families of *S. littoralis*

**Fig. 4:**
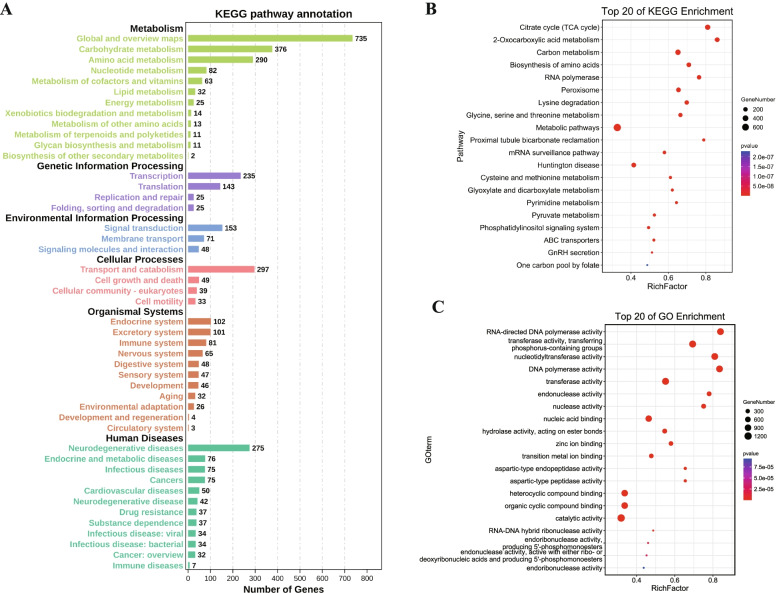
KEGG and GO analysis of rapidly expanded families in *S. littoralis*. **a** Classification and statistics of KEGG pathways of rapidly expanded gene families in *S. littoralis*. **b** KEGG enrichment analysis of rapidly expanded gene families. **c** Functional enrichment of rapidly expanded gene families. RichFactor is the ratio of the number of differential genes in this metabolic pathway to the number of all genes annotated in this pathway. The higher the value, the greater the enrichment degree. The color of the dot in the figure represents *p*-value

CAFE software was used to analyze gene family expansions and contractions. Compared with the common ancestor of *S. littoralis* and *S. litura*, a total of 606 gene families were expanded in *S. littoralis*, while 2784 gene families were contracted (Fig. 3a). Among them, 524 gene families were rapidly evolving families with *P*-values ≤ 0.05. To investigate the function of these rapidly expanded gene families in *S. littoralis*, Kyoto Encyclopedia of Genes and Genomes (KEGG) analysis showed that these families were significantly (*p*-value ≤ 0.05) enriched in carbohydrate metabolism (citrate cycle, glyoxylate and dicarboxylate metabolism), transport and catabolism (peroxisome), signal transduction (phosphatidylinositol signaling system, tumor necrosis factor signaling pathway, the Janus kinase - signal transducers and activators of transcription signaling pathway, etc.), membrane transport (ATP-binding cassette (ABC) transporters) (Fig. 4a and b, Additional file 10: Fig. S3, Additional file 11: Table S8). In addition, endocrine system, excretory system, immune system (Toll-like receptor signaling pathway, NOD-like receptor signaling pathway, etc.), xenobiotic biodegradation and metabolism gene families were also expanded in *S. littoralis*.

Rapid excreting of toxic substances through direct transferring is an important way for insects to avoid the accumulation of toxic substances in their bodies [18, 19]. In the expanded gene families of *S. littoralis*, metabolic pathways, carbohydrate and amino acid metabolism related genes account for a large proportion, which may be associated with the fact that *S. littoralis* feeds on a variety of plants and therefore needs to metabolize a variety of different toxic secondary substances produced by plants. Many of the expanded gene families are involved in the peroxisome pathway. Peroxisome has detoxification function, because the marker enzyme of peroxisome is catalase, which can use H_2_O_2_ to oxidize harmful substances such as phenol, formaldehyde, formic acid and alcohol, converting these toxic substances into non-toxic substances [20]. Phosphatidylinositol signaling pathways are related to an organism’s tolerance to toxic substances. In industrial yeast, *Saccharomyces cerevisiae*, this pathway was reported to be involved in the mediation of yeast tolerance against 5-hydroxymethyl-2-furaldehyde, a commonly encountered toxic compound liberated from lignocellulosic-biomass pretreatment [21]. ABC transporters are a class of transmembrane proteins widely present in organisms, which play an important role in the transport of xenobiotics [22]. The ABC transporters in insects play important roles in plant toxic secondary metabolites transport and pesticide detoxification. All of the pathways mentioned above are likely to be associated with tolerance to plant toxic secondary substances and environmental adaptation in insects.

The result of GO enrichment analysis showed that many expanded gene families were related to the insect’s host or environmental adaptability. For example, in the category of biological processes, the rapidly expanding gene families are involved in heterocycle metabolic, cellular aromatic compound metabolic, organic cyclic compound metabolic, cell wall polysaccharide metabolic, hemicellulose metabolic. In the category of molecular functions, it mainly includes transferase activity, hydrolase activity, catalytic activity, heterocyclic compound binding, organic cyclic compound binding, etc. (Fig. 4c, Additional file 12: Fig. S4, Additional file 13: Fig. S5, Additional file 14: Table S9, Additional file 15: Table S10).

In addition, based on an understanding of the feeding habits of different lepidopteran insects, we classified *B. mori*, *M. sexta* and *P. xylostella* as oligophagous, and *H. armigera*, *T. ni*, *S. exigua*, *S. litura*, *S. frugiperda* and *S. littoralis* as polyphagous. On this basis, it was found that the copy number of gamma-aminobutyric acid type B (GABAB) receptor gene was higher in the polyphagous species (average 7.5 genes) than in the oligophagous species (average 0.67 genes) (Additional file 16: Table S11). GABAB receptor is widely expressed in the nervous system and plays a key role in neuronal excitability [23], which may be related to the perception and response of insects to xenobiotic stimuli such as pesticides and toxic metabolites of plants. Higher GABAB copy number may confer greater adaptability of polyphagous insects to pesticides and different host plants that produce different toxic secondary metabolites.

### Expansion of P450 in *S. littoralis*

The ecological adaptation of pests to different host plants is closely related to their detoxification ability to xenobiotics. Activating the insect’s own ability to metabolize and transport toxins is crucial in this process [22, 24]. In order to elucidate the key genomic changes of *S. littoralis* that have strong adaptability to a variety of host plants and extensive destruction ability, we compared the detoxification associated gene families of four *Spodoptera* species (including *S. littoralis*), *H. armigera*, *M. sexta*. *D. plexippus* and *B. mori*, which is almost monophagous (feeding almost exclusively on mulberry (*Morus* spp.) leaves) (Additional file 17: Table S12). The P450 gene family contains a large number of genes in the polyphagous insects.

**Fig. 5:**
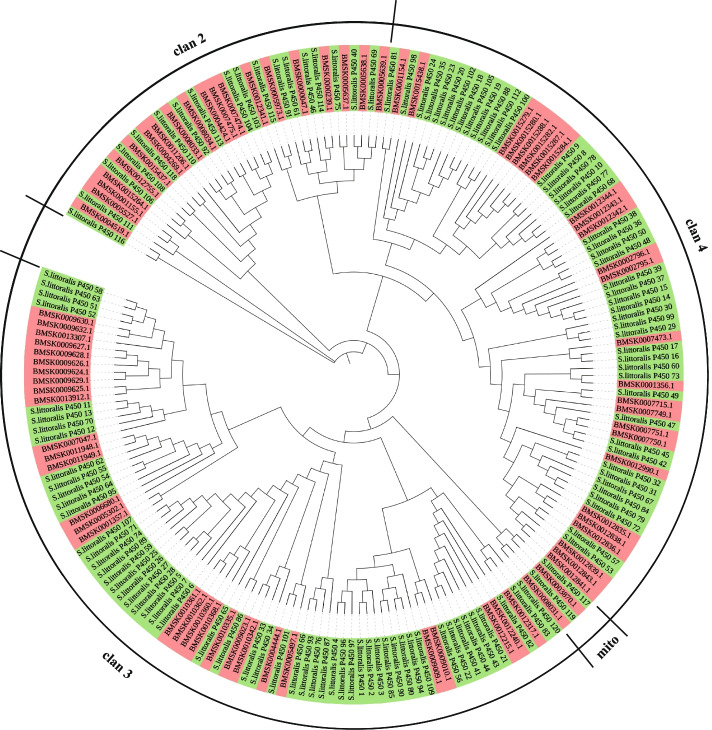
Phylogenetic analysis of P450 genes in *S. littoralis* (green) and *B. mori* (light red). The P450 of *S. littoralis* can be grouped into four clans, and the expansion mainly occurs in clan3 and clan4. The sequences were aligned using MUSCLE. The evolutionary history was inferred using the neighbor-joining method with 1000 bootstrap replicates

Cytochrome P450s make up a superfamily of proteins found in almost all living organisms. Insect P450s are involved in the biotransformation of various xenobiotic compounds, and the enhanced detoxification metabolism mediated by P450s is an important reason for the resistance and cross-resistance of insects to different insecticides [24]. The increase in copy number and up-regulation of P450 gene expression are important mechanisms of enhanced detoxification [25]. In the genome of *S. littoralis*, we identified 120 P450 genes, which were more than that in *B. mori* (90) and *D. plexippus* (76), and also more than that in the important polyphagous agricultural pests *S. exigua* and *H. armigera* (Additional file 17: Table S12). Phylogenetic analysis showed that P450 in clan 3 and clan 4 in *S. littoralis* was expanded compared with *B. mori*, while P450 clan 2 and mito were relatively conserved between the two species (Fig. 5). The expansion phenomenon of P450 clan 3 and clan 4 also occurred in *S. frugiperda* and *S. litura* [12, 26], which are also highly polyphagous.

In the expanded P450 gene families of *S. littoralis*, the duplicated genes were similar to CYP6B1, CYP6B5, CYP6B6, CYP6B7, CYP6A13, CYP4G15 and CYP4C1 of *B. mori*, respectively, and all belong to P450 clan 3 and clan 4. It is reported that CYP4G15 expressed in the brain and central nervous system of *D. melanogaster* [27], and the expression level of CYP4G15 in imidacloprid-resistant Asian *Citrus psyllid* was moderately increased [28]. The tolerance of *Papilio polyxenes* to furocoumarin-rich host plants is attributed to the transcriptional induction of CYP6B1, a gene encoding P450 that metabolizes linear furocoumarins, such as xanthotoxin, at high rates [29]. CYP6B6 is involved in the detoxification of esfenvalerate through hydroxylation in *H. armigera* [30]. The expanded P450 gene family in *S. littoralis* was mostly associated with tolerance to pesticides and toxic plant secondary metabolites. There is a common gene expression pattern between host adaptability and tolerance to multiple insecticides, and several gene families related to detoxification play an important role in this process [31]. The expansion of clan 3 and clan 4 of P450 gene family is strongly correlated with host adaptability and polyphagy of *S. littoralis*, and it is inferred that it also enhances the tolerance of *S. littoralis* to diverse insecticides.

In addition, we compared the expression levels of P450 genes in larvae, pupae, male and female adults of *S. littoralis*. The results showed that several genes of CYP6 subfamily had higher expression levels in larval stage than in other developmental stages (Additional file 18: Fig. S6). CYP6 subfamily is important in insect metabolism of xenobiotics [29, 30]. High expression of these genes is probably also related to the strong host adaptability, polyphagous behavior and insecticide tolerance of *S. littoralis* larvae.

### Expansion of GSTs in *S. littoralis*

**Fig. 6:**
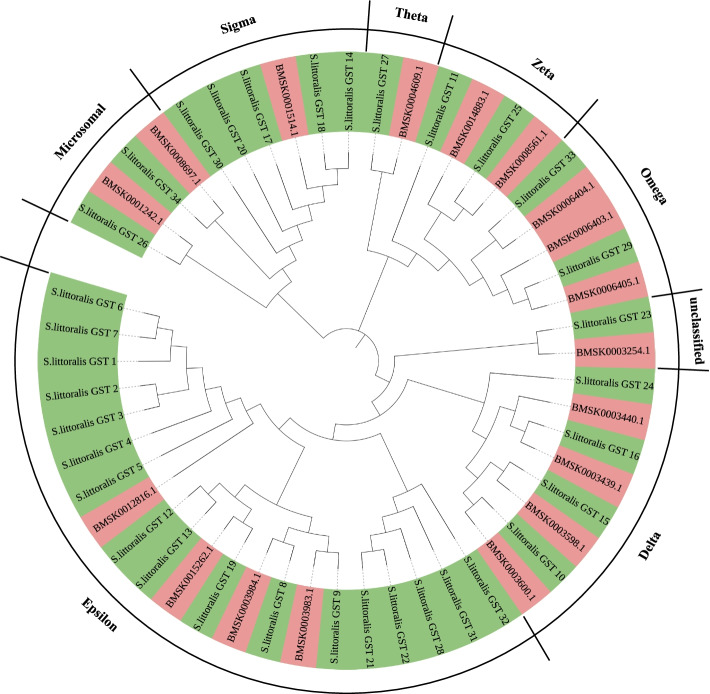
Phylogenetic analysis of GST genes in *S. littoralis* (green) and *B. mori* (light red). The GST gene family was grouped into eight classes, epsilon and sigma GST classes were expanded in *S. littoralis* genome. The sequences were aligned using MUSCLE. The evolutionary history was inferred using the neighbor-joining method with 1000 bootstrap replicates

Glutathione S transferases (GSTs), which catalyze the binding of nucleophilic glutathione to various electron-friendly exogenous chemicals, play an important role in the detoxification of xenobiotic compounds and are related to insecticide tolerance [32]. There were 34 GST genes identified in the *S. littoralis* genome, the number of GST genes in *B. mori* and *D. plexippus* genomes was only 26 and 24, respectively (Additional file 17: Table S12). In the four other polyphagous insect species we analyzed, the number of GST genes ranged from 33 to 39, excluding *S. frugiperda* (58). The phylogenetic tree showed that epsilon and sigma GST classes were expanded in the *S. littoralis* genome compared to *B. mori* (Fig. 6). Transcriptomic analysis of different developmental stages showed that the expression level of most epsilon GSTs in larval stages was higher than that in pupal and adult stage (Additional file 19: Fig. S7).

The epsilon GST class was closely related to the tolerance to dichlorodiphenyltrichloroethane (DDT) and pyrethroid insecticides in *Aedes aegypti* [33]. In *Nilaparvata lugens*, the epsilon GST class could recognize insecticides as substrates, thus playing a key role in xenobiotic detoxification [34]. In *Argopecten irradians*, sigma class GST is a fragile but efficient antioxidant enzyme, which was potentially involved in the innate immune responses [35]. Sigma class of GST also involved in carbaryl detoxification in *Locusta migratoria manilensis* [36]. In *S. littoralis*, epsilon and sigma GST class are likely to be involved in the detoxification of insecticides and other xenobiotics. In Egypt, 4,4′- DDT and Carvinine were less effective against *S. littoralis* [37], which may be related to the expansion of GST gene family in epsilon and sigma classes.

### Other detoxification-related gene families

In addition to P450 and GST, we also analyzed other gene families closely associated with detoxification metabolism in insects. A total of 46 UDP-glucuronosyltransferase (UGT) genes, 14 protein tyrosine phosphatase genes, 3 patched domain containing genes, 53 ABC transporter genes and 71 carboxyl/choline esterase (CCE) genes were identified in *S. littoralis* (Additional file 17: Table S12). Compared with other insects of genus Spodoptera or even Lepidoptera, these gene families did not expand significantly. However, they also play a key function in detoxification metabolism in *S. littoralis*. For example, nearly half of the genes in the CCE family are significantly more expressed at larval stage than at other developmental stages (Additional file 20: Fig. S8). The ABC transporter genes *ABCA2* (*SL14310*), *ABCB1* (*SL07686*), *ABCC2* (*SL07640*), *ABCC3* (*SL07639*), and *ABCC4* (*SL011756*), which are closely related to xenobiotic transport [22], likewise exhibit the highest expression levels in the larval stage in *S. littoralis* (Additional file 21: Fig. S9).

### Chemosensory gene families of *S. littoralis*

Chemoreception is an important biological process in insects, which is closely related to a series of physiological activities such as foraging, mating, oviposition and avoiding natural enemies. By comparing chemoreception-related gene families in *S. littoralis*, *H. armigera*, *M. sexta*, *D. plexippus*, *B. mori* and three other *Spodoptera* species, we found that the number of gustatory receptor (GR) genes in the *S. littoralis* genome was 256, much more than that in *B. mori* (76), *D. plexippus* (47) and *M. sexta* (65) genome (Additional file 17: Table S12, Additional file 22 Fig. S10). In addition, four *Spodoptera* species (including *S. littoralis*) and *H. armigera* had a large number of GR genes (ranging from 186 to 256). The number of GR genes in the genomes of *Heliothis virescens* and *T. ni* reached or approached 200 [38], and both of them also belonging to Noctuidae. Therefore, it is reasonable to speculate that there may be specific expansions of GR gene families during the evolution of Noctuidae polyphagous species, which may be related to their feature of wide host plant range. GRs are closely related to insect feeding activities, the expansion of GR gene family may be beneficial for the Noctuidae species to distinguish different hosts and obtain sufficient food.

Odorant binding proteins (OBP) and odorant receptors (OR) are critical genes in insect recognition of volatiles emitted by plants. In *S. littoralis*, the number of these two gene families is 39 and 69 respectively, which is slightly less than that in *S. frugiperda* and *S. litura*. This could be due to the fact that this species’ migratory and flying abilities are less developed than those of the other two closely related species. Migrating insects need to identify volatiles of many different plants to accurately locate their host plants, and these two chemosensory gene families may be involved in the recognition of plant volatiles. Transcriptome analysis of larvae, pupae, female adults and male adults showed that there was no significant difference in the expression level of OR genes at different developmental stages, while multiple OBP genes were highly expressed in male adult (Additional file 23: Fig. S11 and Additional file 24: Fig. S12). The high expression of some OBP genes in male adults may be related to courtship behavior of *S. littoralis*, which may play a role in recognizing the sex pheromone released by female adults.

In *S. littoralis*, the number of chemosensory proteins (CSP), ionotropic receptors (IR) and sensory neuron membrane proteins (SNMP) genes was 20, 55 and 12, respectively. There was little difference in the number of these gene families among the species used for comparison.

## Conclusion

Through genome sequencing and comparative genomics, we reveal the genome-level reasons for the polyphagy of *S. littoralis*, a major pest of the genus *Spodoptera*. The divergence time of *S. littoralis*, *S. litura* and *S. frugiperda* was relatively recent. The significantly expanded gene families are mainly involved in xenobiotics transport and metabolism. Several gene families related to detoxification and chemosensation were significantly expanded, such as P450, GST, GR, etc. The expansion of these gene families is the result of adaptive evolution in the species. Due to the differences in secondary metabolites produced by different host plants, the expansion of gene families related to detoxification and chemosensation is the basis for pest to recognize and feed on a variety of plants. Thus, evidence of the expansion of these gene families suggests that *S. littoralis* has a wide range of host plants and environmental adaptability characteristic at the genomic level, which is consistent with reports of risk to crops and vegetables [8]. Based on the common gene expression pattern of host adaptation and pesticide tolerance [31], it was speculated that the expansion of detoxification related gene families, such as P450 and GST, could not only improve host plant adaptability and polyphagy, but also enhance the tolerance of *S. littoralis* to different pesticides. So that they can evolve more rapidly into insecticide-resistant populations, bringing more challenges to the management of this pest. The expansion of GR gene family is also an important feature of *S. littoralis* and even the polyphagous insects of Noctuidae.

This study is the first to reveal the genomic characteristics of *S. littoralis* as a highly polyphagous organism. In addition, this work provides high-quality genomic resources for subsequent genome-wide studies of other biological problems in *S. littoralis*. The combination of genomics and biological characteristics will help us to further understand the molecular mechanisms of pest damage to crops, and provide a theoretical basis for developing new pest management strategies and controlling major agricultural pests more scientifically.

## Methods

### Insects

One male moth of *S. littoralis* was selected for genome sequencing. The sample was from an inbred strain which was established with individuals collected in Egypt in 2011, then formed after successive generations of laboratory rearing at Lancaster University, UK. Larvae were reared in the laboratory using standard semi-artificial wheatgerm-based diet at 26 °C with a 14:10, light:dark photoperiod. Adult moths were provided with 5% sugar water [39].

### Genome sequencing

Genomic DNA was extracted using the Qiagen Genomic DNA kit (Cat. no. 13323, Qiagen). DNA purity and quantification were determined with a NanoDrop One UV-Vis spectrophotometer (Thermo Fisher Scientific) and Qubit 3.0 Fluorometer (Invitrogen), respectively. A total of 5 μg genomic DNA (gDNA) was used as an input for ~ 20-kb insert libraries (SMRT bell Template Prep Kit 1.0, PacBio) sequenced on the PacBio Sequel (Pacific Biosciences). About 0.5 μg gDNA from the same individual was used as input to generate a 350-bp PCR-free library using the Truseq Nano DNA HT Sample preparation Kit (Illumina), which was then sequenced using the Illumina NovaSeq 6000 platform with 150-bp paired-end reads.

### Genome assembly and evaluation

The Illumina raw reads were filtered by trimming the adapter and low-quality regions using clean_adapter version 1.1 with the parameter “-a Both-adapter -r 75 -s 12” and clean_lowqual version 1.0 with the parameter “-e 0.01 -r 75” (https://github.com/fanagislab/assembly_2ndGeneration/tree/master/clean_illumina). Before the genome assembly, we counted the k-mer frequency from the filtered Illumina reads by using kmerfreq version 4.0 (https://github.com/fanagislab/kmerfreq) [40] with the parameter “-k 17 -t 20 -m 1 -q 5”. The PacBio raw reads were assembled to contigs by Canu version 1.9 (https://github.com/marbl/canu) [41]. Initial contigs were processed by PURGE_DUPS version 1.2.3 (https://github.com/dfguan/purge_dups) using default settings to remove potential heterozygous sequences. Finisher_SC version 2.1 (https://github.com/kakitone/finishingTool) [42] was used to correct assembly errors using PacBio raw reads. The filtered Illumina reads were aligned to the assembled sequences by BWA-MEM version 0.7.5a-r405 (http://bio-bwa.sourceforge.net/bwa.shtml) [43], and then the sequences were polished by PILON version 1.23 (https://github.com/broadinstitute/pilon) [44]. Finally, the sequences was constructed into scaffolds by SSPACE version 3.0 [45].

We evaluated the completeness of the assembled genome sequence in *S. littoralis* using BUSCO version 4 [46] software with insecta_odb10 data sets under default parameters.

### Transcriptome sequencing and analysis

Three fifth-instar larvae, three pupae, three female moths and three male moths from the same inbred strain were used for RNA sequencing. Total RNA was extracted using the RNeasy Mini extraction kit (Qiagen), and a NanoPhotometer spectrophotometer (Implen) and Qubit 2.0 Flurometer (Life Technologies) were used to check the purity and concentration of RNA, respectively. One microgram total RNA per sample was used to make indexed cDNA libraries using the NEBNext Ultra RNA Library Prep Kit for Illumina (NEB) following the manufacturer’s recommendations. The libraries had insert sizes of 250–300 bp and were sequenced on the Illumina NovaSeq 6000 platform with 150-bp paired-end output.

Low-quality bases in the RNA-Seq raw reads were first filtered using clean_adapter version 1.1 and clean_lowqual version 1.0 with default parameters. The clean reads were aligned to the assembled genome using HISAT2 version 2.0.5 (https://github.com/infphilo/hisat2) [47]. To determine gene expression levels in different development stages, RNA-Seq alignments were assembled into putative transcripts by STRINGTIE version 1.3.3b (https://github.com/gpertea/stringtie) [48], and differentially expressed genes were identified by DESEQ2 [49].

### Genome annotation

A de novo repeat library was constructed by RepeatModeler version 1.0.11 (http://www.repeatmasker.org/RepeatModeler.html). Transposable elements (TEs) were identified by RepeatMasker version 4.0.7 (http://www.repeatmasker.org/) using both the de novo library and Repbase library (Repbase - 20170127), and tandem repeats were predicted using tandem repeats finder (TRF) version 4.09 [50]. The repeat sequences of several other Lepidoptera species were analyzed using the same method and compared with the repeat elements of *S. littoralis*.

Gene prediction was performed using homology-based, RNAseq based and de novo methods. We performed ab initio prediction using AUGUSTUS version 3.2.2 with default parameters [51]. For homology-based prediction, we used non-redundancy protein sequences from three *Spodoptera* species (*S. exigua*, *S. frugiperda* and *S. litura*) to align to the *S. littoralis* genome sequences by GENBLASTA version 1.0.4 with default parameters [52]. GENEWISE version 2.4.1 [53] was used to predict the gene models by the GENBLASTA results. For the RNA-seq prediction, the RNA-seq data were mapped to the assembled genome using TOPHAT version 2.0.12 and alignments were processed by CUFFLINKS version 2.2.1 with default parameters to generate transcript predictions [54]. The gene models were predicted by EVidence Modeler version 1.1.1 [55], embedded in a pipeline that integrates evidence from ab initio predictions, homology-based searches, and RNA-seq alignments. Then, the protein-coding sequences were mapped by RNA-seq data and functionally annotated using UniProt [56] and KEGG databases [57]. Finally, the gene models were retained if they had at least one supporting evidence.

Gene functional annotation was performed by aligning the protein sequences to UniProt, Nr, eggNOG and KEGG databases using BLASTP v2.3.0+ (http://blast.ncbi.nlm.nih.gov/Blast.cgi) with E-value cutoff of 10^− 5^. The pathway analysis and functional classification were conducted based on KEGG database.

### Phylogenetic analysis

Thirteen species were selected for the identification of orthologous and paralogous gene families, including *S. littoralis* (this study), *B. mori* (GCF_000151625.1, from NCBI), *D. plexippus* (GCF_009731565.1, from NCBI), *D. melanogaster* (GCF_000001215.4, from NCBI), *H. melpomene* (from Ensembl, http://metazoa.ensembl.org/), *H. armigera* (GCF_002156985.1, from NCBI), *M. sexta* (GCF_014839805.1, from NCBI), *P. xuthus* (GCF_000836235.1, from NCBI), *P. xylostella* (GCF_000330985.1, from NCBI), *S. exigua* (GCA_015679615.1, from NCBI), *S. frugiperda* (from InsectBase, http://www.insect-genome.com), *S. litura* (GCA_002706865.2, from NCBI) and *T. ni* (GCF_003590095.1, from NCBI). Before phylogenetic analysis, redundant data was removed from the sequences of the above mentioned species, and only one of the longest protein sequences is retained for each gene.

Orthology analysis was performed following the pipeline of Orthofinder version 2.4.0 with default parameters. in which, DIAMOND software was utilized for sequence alignment and Markov Cluster Algorithm was used for orthogroup clustering. Single-copy homologous protein sequences from the 13 species were aligned by MUSCLE version 3.8.31 [58], then conserved sequences were extracted using Gblocks version 0.91b with parameters ‘-b4 = 5 -b5 = h -t = p -e = .2’, and each sequence file was sorted and converted from multi-line sequences to one-line sequences using SeqKit version 0.14.0 [59]. Finally, all single-copy sequences of each species were merged into one-line by ‘paste’ command. ProtTest version 3.4 was used to predict the optimal amino acid substitution model, RAxML-NG (random axelerated maximum likelikhood - Next Generation) version 1.0.2 [60] was used to construct phylogenetic tree using the optimal model with 1000 bootstrap replicates. Divergence time of *D. melanogaster* and *B. mori* (243–317 million years ago), *B. mori* and *H. armigera* (99–121 million years ago) were obtained from the timetree website (http://timetree.org/), and then used as two calibrations to predict divergence times between other species. The MCMCtree in PAML v4.9j [61] was used to evaluate the divergence time based on the topology of the phylogenetic tree with the parameters of ‘seqtype = 2 usedata = 3 clock = 2 RootAge <= 3.0 alpha = 0.5’. The result was visualized using FigTree version 1.4.4 (http://tree.bio.ed.ac.uk/software/figtree/).

### Whole genome synteny

Whole-genome synteny was estimated between *S. littoralis*, *S. litura*, and *B. mori*. Firstly, protein sequence alignment between different species was performed using BLASTP (BLAST version 2.6.0) (E-value <1E−10). Then, the synteny blocks were analyzed using MCScanX software with default parameters.

### Gene family expansion and contraction

In addition, the gene family expansion and contraction were analyzed using CAFE version 4.2 [62] based on the ultrametric tree extracted by r8s and the result of orthology analysis. We used a criterion of *p* < 0.05 for significantly changed gene families. The KEGG and GO enrichment analysis were conducted on the significant expanded families in *S. littoralis* using the OmicShare tools, a free online platform for data analysis (www.omicshare.com/tools).

### Gene family analysis

In this study, we annotated the gene families related to host plant adaptation in detoxification and chemoreception, including cytochrome P450 (P450), glutathione S-transferase (GST), UDP-glucuronosyltransferase (UGT), chemosensory proteins (CSPs), odorant binding proteins (OBPs), olfactory receptors (ORs), gustatory receptors (GRs), etc.

To identify the GR gene family, NCBI GeneBank, InsectBase and other database were used to collect GR protein sequences of lepidopteran insects, especially related species of *S. littoralis*, and TBLASTN (BLAST version 2.6.0) was used to search the *S. littoralis* genome. Then, GENEWISE v2.4.1 was used to define the gene structure and obtain candidate GR protein sequences of *S. littoralis*. The same analysis strategy was adopted for other species used for comparison. STRINGTIE v1.3.3b was used for GR gene expression level analysis.

To identify the P450, GST, OR and other gene families in *S. littoralis*, the published insect protein sequences of each gene family were collected from NCBI GenBank, InsectBase and other database. Then the sequences were manually confirmed to obtain the cleaned reference protein sequences of each gene families. Then, BLASTP (BLAST version 2.6.0) (E-value <1E−5) was used to determine candidate sequences in *S. littoralis* by comparing with cleaned reference sequences. Identified genes were further examined by HMMER3 search (cutoff E-value <1E−5) using the Pfam database to confirm conserved domains in each gene family [63]. For supplement and validation, information from gene function annotations was utilized.

Phylogenetic trees for each gene family was constructed by amino acid sequences. The software MUSCLE was used to construct the multiple alignment. Then a neighbor-joining (NJ) evolutionary history was inferred using MEGA-X with 1000 bootstrap replicates. Positions with less than 95% site coverage were eliminated [22].

## Supplementary Information

**Additional file 1.**

**Additional file 2.**

**Additional file 3.**

**Additional file 4.**

**Additional file 5.**

**Additional file 6.**

**Additional file 7.**

**Additional file 8.**

**Additional file 9.**

**Additional file 10.**

**Additional file 11.**

**Additional file 12.**

**Additional file 13.**

**Additional file 14.**

**Additional file 15.**

**Additional file 16.**

**Additional file 17.**

**Additional file 18.**

**Additional file 19.**

**Additional file 20.**

**Additional file 21.**

**Additional file 22.**

**Additional file 23.**

**Additional file 24.**

## Data Availability

The Whole Genome Shotgun project has been deposited at DDBJ/ENA/GenBank under the accession JAIPRO000000000. The version described in this paper is version JAIPRO010000000.1 (https://www.ncbi.nlm.nih.gov/nuccore/JAIPRO000000000.1/). Raw sequencing reads of PacBio, Illumina and RNA-seq in this paper have been deposited in the NCBI Sequence Read Archive (SRA) under BioProject accession number PRJNA760296 (https://www.ncbi.nlm.nih.gov/sra/?term=PRJNA760296). Genome assembly have been deposited in the NCBI with the BioProject accession number PRJNA759792 and BioSample Accession SAMN21197648. The genome assembly and annotation files are available at the website ftp://ftp.agis.org.cn/Spodoptera_littoralis.
