# Supplementary material for: Genomic features of the polyphagous cotton leafworm Spodoptera littoralis
Source: BMC Genomics. 2022 May 7;23:353. doi: 10.1186/s12864-022-08582-w (PMC9080191; doi:10.1186/s12864-022-08582-w)
Supplement: Supplementary file 1 — Additional file 1. [file 12864_2022_8582_MOESM1_ESM.docx]

Additional file 1: Table S1. Statistics of genomic sequencing data.

| Sequencing platform | Insert size (bp) | Total clean reads | Total clean bases | Sequence coverage (×) |
| --- | --- | --- | --- | --- |
| Illumina | 350 | 1105312022 | 164517738872 | 376 |
| PacBio | ~20000 | 4447934 | 55178979906 | 126 |
