# Supplementary material for: Genomic features of the polyphagous cotton leafworm Spodoptera littoralis
Source: BMC Genomics. 2022 May 7;23:353. doi: 10.1186/s12864-022-08582-w (PMC9080191; doi:10.1186/s12864-022-08582-w)
Supplement: Supplementary file 2 — Additional file 2. [file 12864_2022_8582_MOESM2_ESM.docx]

Additional file 2: Table S2. Basic information about the assembled genome of *S. littoralis*.

| Items | Contig Number | Contig Length (bp) | Scaffold Number | Scaffold Length (bp) |
| --- | --- | --- | --- | --- |
| Total | 308 | 436841583 | 238 | 436555369 |
| Maximum Length | 1 | 15838663 | 1 | 15972555 |
| Minimum Length | 1 | 1936 | 1 | 1936 |
| Average Length | - | 1418320 | - | 1834270 |
| Median Length | - | 429940 | - | 581467 |
| N10 Length | 3 | 13112852 | 3 | 15852893 |
| N20 Length | 7 | 9641413 | 6 | 14623917 |
| N30 Length | 13 | 6779027 | 10 | 9641309 |
| N40 Length | 20 | 5904519 | 15 | 7494814 |
| N50 Length | 29 | 4356875 | 22 | 6091645 |
| N60 Length | 40 | 3187498 | 31 | 4424391 |
| N70 Length | 56 | 2291425 | 43 | 2930051 |
| N80 Length | 79 | 1387182 | 61 | 2009683 |
| N90 Length | 118 | 760662 | 91 | 1172647 |
