# Supplementary figures and images for: Genomic features of the polyphagous cotton leafworm Spodoptera littoralis
Source: BMC Genomics. 2022 May 7;23:353. doi: 10.1186/s12864-022-08582-w (PMC9080191; doi:10.1186/s12864-022-08582-w)

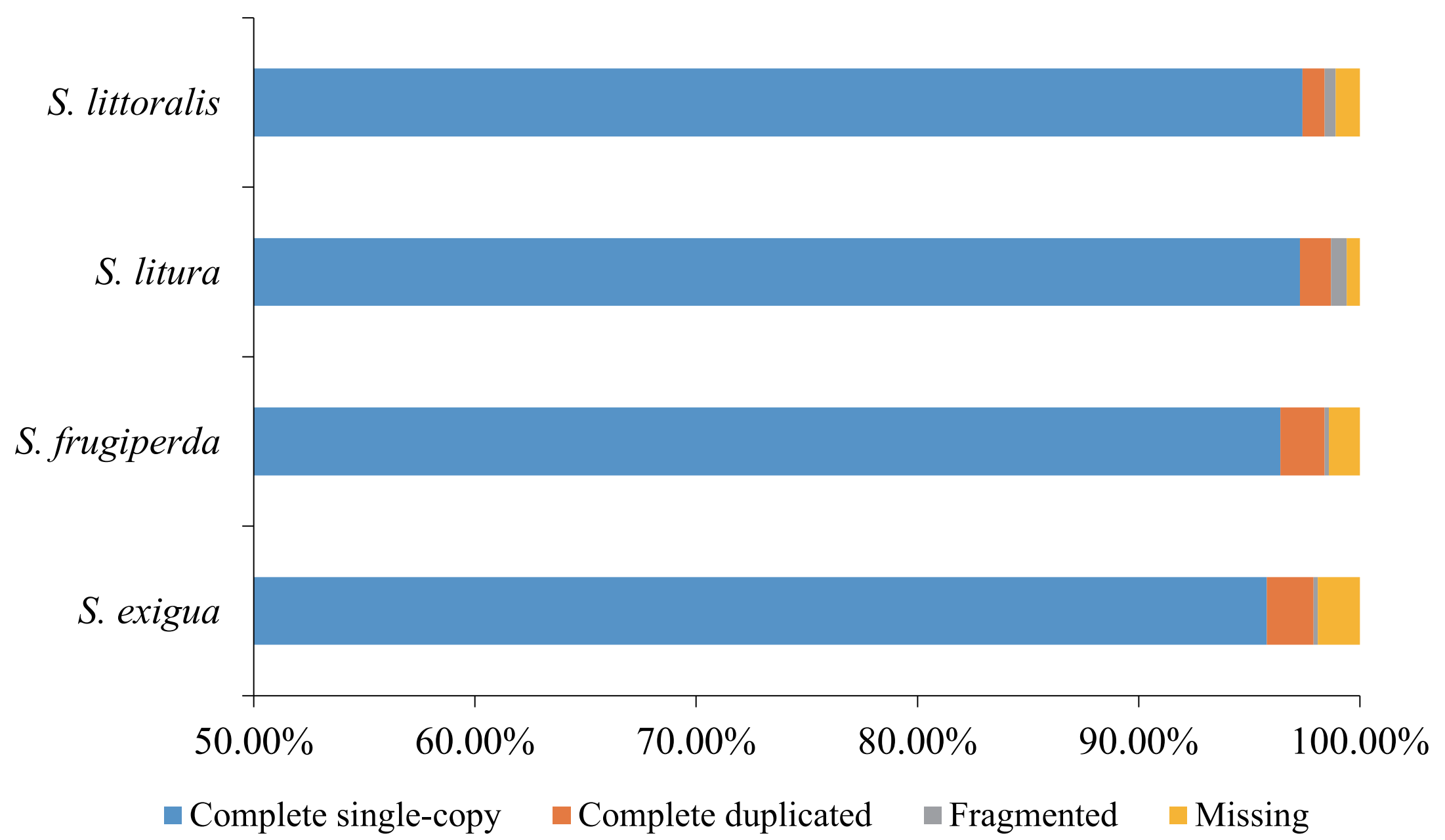

**Additional file 3: Fig. S1.** The assembled genome quality was evaluated using BUSCO software

Supplement: Supplementary file 3 — Additional file 3. [file 12864_2022_8582_MOESM3_ESM.pdf]

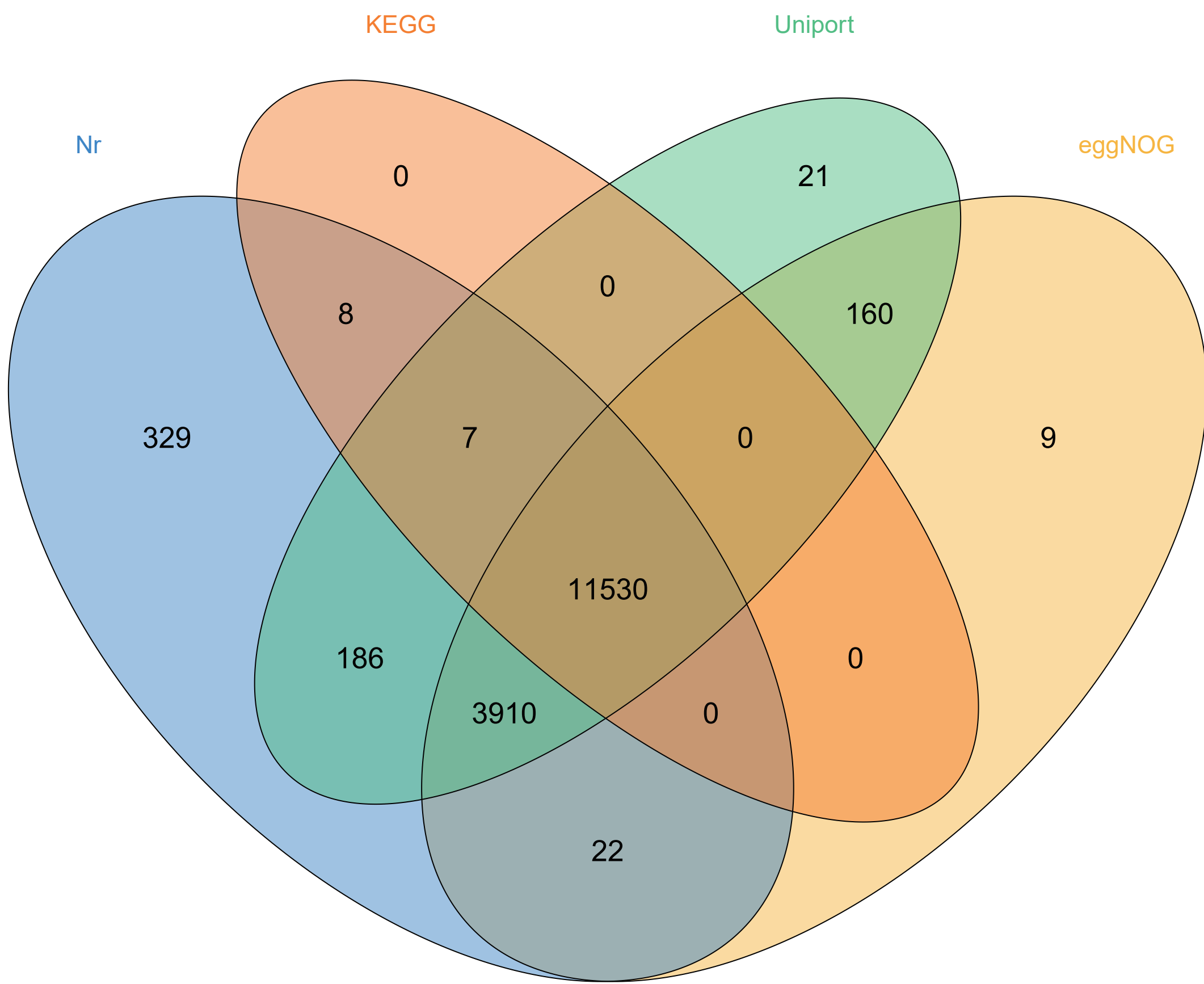

**Additional file 5: Fig. S2.** Functional annotation of *S. littoralis* predicted genes using 4 databases

Supplement: Supplementary file 5 — Additional file 5. [file 12864_2022_8582_MOESM5_ESM.pdf]

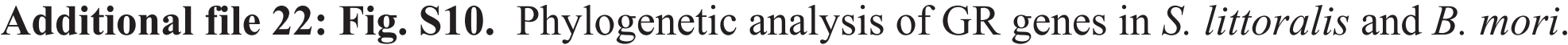

Supplement: Supplementary file 22 — Additional file 22. [file 12864_2022_8582_MOESM22_ESM.pdf]
