# Supplementary material for: Genomic features of the polyphagous cotton leafworm Spodoptera littoralis
Source: BMC Genomics. 2022 May 7;23:353. doi: 10.1186/s12864-022-08582-w (PMC9080191; doi:10.1186/s12864-022-08582-w)
Supplement: Supplementary file 4 — Additional file 4. [file 12864_2022_8582_MOESM4_ESM.docx]

Additional file 4: Table S3. Annotation and distribution of repetitive elements in *S. littoralis* genome.

| Family | No. of Repeat | Length(bp) | Percent (%) |
| --- | --- | --- | --- |
| DNA: | 33303 | 8452801 | 1.9362495 |
| DNA unknown: | 8 | 392 | 0.0000898 |
| LINE: | 197184 | 53673844 | 12.2948537 |
| LTR: | 61690 | 16382903 | 3.7527664 |
| Low_complexity: | 11715 | 551199 | 0.1262610 |
| Other: | 1 | 64 | 0.0000147 |
| RC: | 109128 | 18979534 | 4.3475663 |
| RC unknown: | 1 | 68 | 0.0000156 |
| Retroposon: | 182 | 29226 | 0.0066947 |
| Retroposon unknown: | 4 | 277 | 0.0000635 |
| SINE: | 57033 | 11699744 | 2.6800138 |
| SINE unknown: | 98 | 16618 | 0.0038066 |
| Satellite: | 1166 | 161882 | 0.0370817 |
| Simple_repeat: | 85462 | 5676245 | 1.3002348 |
| Unknown: | 279229 | 47074050 | 10.7830652 |
| Unspecified-Unknown: | 1 | 40 | 0.0000092 |
| begin-Unknown: | 1 | 1 | 0.0000002 |
| scRNA-Unknown: | 1 | 234 | 0.0000536 |
| rRNA: | 83 | 37443 | 0.0085769 |
| snRNA: | 157 | 18365 | 0.0042068 |
| tRNA: | 268 | 20675 | 0.0047359 |
| Total: | 836715 | 162775605 | 37.2863597 |
