# Supplementary material for: Genomic features of the polyphagous cotton leafworm Spodoptera littoralis
Source: BMC Genomics. 2022 May 7;23:353. doi: 10.1186/s12864-022-08582-w (PMC9080191; doi:10.1186/s12864-022-08582-w)
Supplement: Supplementary file 6 — Additional file 6. [file 12864_2022_8582_MOESM6_ESM.docx]

Additional file 6: Table S4. Statistics on functional annotation of *S. littoralis* predicted genes.

| Item | Gene number | Percent(%) |
| --- | --- | --- |
| Total | 17207 | 100.00 |
| Annotated: | 16182 | 94.04 |
| Nr | 15992 | 92.94 |
| eggNOG | 15631 | 90.84 |
| KEGG | 11545 | 67.09 |
| TrEMBL | 15814 | 91.90 |
| Unannotated | 1025 | 5.96 |
