# Supplementary material for: Genomic features of the polyphagous cotton leafworm Spodoptera littoralis
Source: BMC Genomics. 2022 May 7;23:353. doi: 10.1186/s12864-022-08582-w (PMC9080191; doi:10.1186/s12864-022-08582-w)
Supplement: Supplementary file 7 — Additional file 7. [file 12864_2022_8582_MOESM7_ESM.docx]

Additional file 7: Table S5. Summary of orthogroups in thirteen insects.

| Species | Total genes | Genes in orthogroups | Unassigned genes | Orthogroups containing species | Species specific orthogroups | Species specific genes | Ave.genes per orthogroups |
| --- | --- | --- | --- | --- | --- | --- | --- |
| *S.littoralis* | 17207 | 16947 | 260 | 10292 | 158 | 1544 | 1.65 |
| *S.litura* | 16182 | 15573 | 609 | 12144 | 101 | 292 | 1.28 |
| *S.frugiperda* | 18811 | 18567 | 244 | 11492 | 73 | 321 | 1.62 |
| *S.exigua* | 14503 | 13730 | 773 | 10161 | 133 | 572 | 1.35 |
| *H.armigera* | 13836 | 13628 | 208 | 11230 | 25 | 80 | 1.21 |
| *T.ni* | 15089 | 14810 | 279 | 11194 | 45 | 175 | 1.32 |
| *B.mori* | 13800 | 13453 | 347 | 10818 | 60 | 247 | 1.24 |
| *M.sexta* | 15967 | 15646 | 321 | 11141 | 123 | 550 | 1.40 |
| *D.plexippus* | 13105 | 12862 | 243 | 10688 | 49 | 211 | 1.20 |
| *H.melpomene* | 12829 | 11954 | 875 | 9909 | 64 | 162 | 1.21 |
| *P.xuthus* | 15131 | 14622 | 509 | 11378 | 818 | 2253 | 1.29 |
| *P.xylostella* | 18119 | 17082 | 1037 | 10397 | 307 | 992 | 1.64 |
| *D.melanogaster* | 13968 | 11353 | 2615 | 8103 | 473 | 1904 | 1.40 |
