# Supplementary material for: Genomic features of the polyphagous cotton leafworm Spodoptera littoralis
Source: BMC Genomics. 2022 May 7;23:353. doi: 10.1186/s12864-022-08582-w (PMC9080191; doi:10.1186/s12864-022-08582-w)
Supplement: Supplementary file 8 — Additional file 8. [file 12864_2022_8582_MOESM8_ESM.docx]

Additional file 8: Table S6. Genomic comparison of thirteen sequenced species.

| Species | 1:1:1 orthologs | N:N:N orthologs | Lepidoptera specific | Noctuidae specific | Spodoptera specific | Speciesspecific | Other orthologs | Unclustered genes |
| --- | --- | --- | --- | --- | --- | --- | --- | --- |
| *S.littoralis* | 1170 | 3842 | 1584 | 71 | 145 | 1544 | 8591 | 260 |
| *S.litura* | 1170 | 4134 | 1732 | 98 | 120 | 292 | 8027 | 609 |
| *S.frugiperda* | 1170 | 5409 | 2199 | 128 | 163 | 321 | 9177 | 244 |
| *S.exigua* | 1170 | 4021 | 1577 | 73 | 128 | 572 | 6189 | 773 |
| *H.armigera* | 1170 | 4093 | 1719 | 86 | - | 80 | 6480 | 208 |
| *T.ni* | 1170 | 4406 | 1745 | 106 | - | 175 | 7208 | 279 |
| *B.mori* | 1170 | 4154 | 1623 | - | - | 247 | 6259 | 347 |
| *M.sexta* | 1170 | 4779 | 1890 | - | - | 550 | 7257 | 321 |
| *D.plexippus* | 1170 | 4135 | 1605 | - | - | 211 | 5741 | 243 |
| *H.melpomene* | 1170 | 4133 | 1616 | - | - | 162 | 4873 | 875 |
| *P.xuthus* | 1170 | 3910 | 1532 |  | - | 2253 | 5757 | 509 |
| *P.xylostella* | 1170 | 5839 | 2065 | - | - | 992 | 7016 | 1037 |
| *D.melanogaster* | 1170 | 4351 | - | - | - | 1904 | 3928 | 2615 |
