# Supplementary material for: Genomic features of the polyphagous cotton leafworm Spodoptera littoralis
Source: BMC Genomics. 2022 May 7;23:353. doi: 10.1186/s12864-022-08582-w (PMC9080191; doi:10.1186/s12864-022-08582-w)
Supplement: Supplementary file 9 — Additional file 9. [file 12864_2022_8582_MOESM9_ESM.docx]

Additional file 9: Table S7. The synteny analysis of *S .littoralis*, *S. litura* and *B. mori*.

| Synteny | Blocks (≥10 genes) | |  | Blocks (≥5 genes) | |
| --- | --- | --- | --- | --- | --- |
|  | Block number | Gene number of S. littoralis |  | Block number | Gene number of S. littoralis |
| S. littoralis - S. litura | 289 | 8252 |  | 390 | 8923 |
| S. littoralis - B. mori | 175 | 7031 |  | 259 | 7564 |
