# Supplementary material for: Genomic features of the polyphagous cotton leafworm Spodoptera littoralis
Source: BMC Genomics. 2022 May 7;23:353. doi: 10.1186/s12864-022-08582-w (PMC9080191; doi:10.1186/s12864-022-08582-w)
Supplement: Supplementary file 10 — Additional file 10. [file 12864_2022_8582_MOESM10_ESM.pdf]

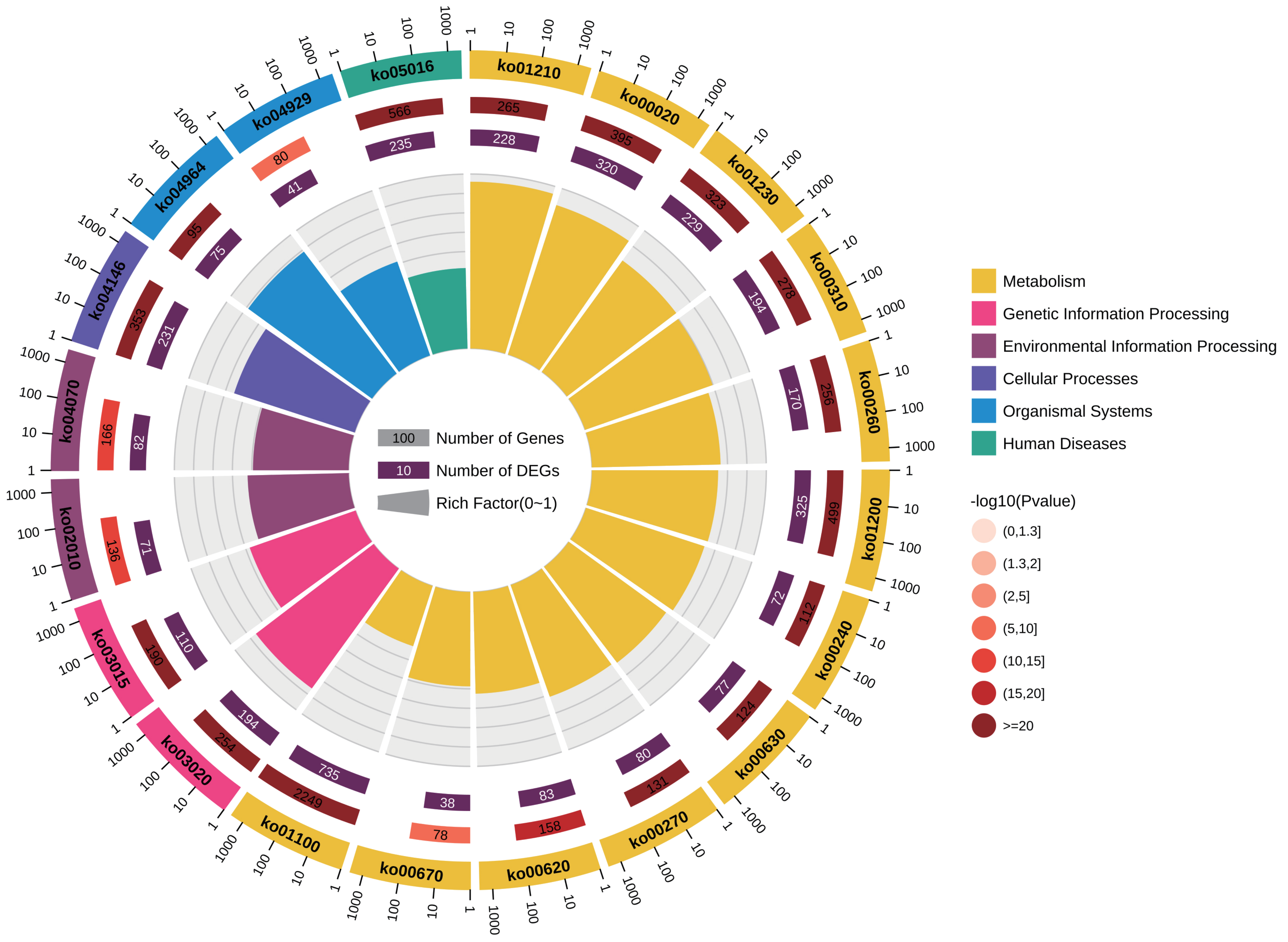

Additional file 10: Fig. S3. Kegg enrichment pathways of rapidly expanded gene families in *S. littoralis*
