# Supplementary material for: Genomic features of the polyphagous cotton leafworm Spodoptera littoralis
Source: BMC Genomics. 2022 May 7;23:353. doi: 10.1186/s12864-022-08582-w (PMC9080191; doi:10.1186/s12864-022-08582-w)
Supplement: Supplementary file 11 — Additional file 11. [file 12864_2022_8582_MOESM11_ESM.docx]

Additional file 11: Table S8. Kegg enrichment analysis on *S. littoralis* rapidly expanded gene families (top 50)

| KEGG_A_class | KEGG_B_class | Pathway | out (1535) | All (6871) | Pvalue | Qvalue | Pathway ID |
| --- | --- | --- | --- | --- | --- | --- | --- |
| Metabolism | Carbohydrate metabolism | Citrate cycle (TCA cycle) | 320 | 395 | 6.46E-146 | 1.69E-143 | ko00020 |
| Metabolism | Global and overview maps | 2-Oxocarboxylic acid metabolism | 228 | 265 | 2.49E-113 | 3.25E-111 | ko01210 |
| Metabolism | Global and overview maps | Carbon metabolism | 325 | 499 | 7.12E-102 | 6.20E-100 | ko01200 |
| Metabolism | Global and overview maps | Biosynthesis of amino acids | 229 | 323 | 1.41E-81 | 9.22E-80 | ko01230 |
| Genetic Information Processing | Transcription | RNA polymerase | 194 | 254 | 4.19E-78 | 2.19E-76 | ko03020 |
| Cellular Processes | Transport and catabolism | Peroxisome | 231 | 353 | 1.80E-71 | 7.83E-70 | ko04146 |
| Metabolism | Amino acid metabolism | Lysine degradation | 194 | 278 | 7.68E-67 | 2.86E-65 | ko00310 |
| Metabolism | Amino acid metabolism | Glycine, serine and threonine metabolism | 170 | 256 | 1.65E-53 | 5.39E-52 | ko00260 |
| Metabolism | Global and overview maps | Metabolic pathways | 735 | 2249 | 4.08E-45 | 1.18E-43 | ko01100 |
| Organismal Systems | Excretory system | Proximal tubule bicarbonate reclamation | 75 | 95 | 5.26E-32 | 1.37E-30 | ko04964 |
| Genetic Information Processing | Translation | mRNA surveillance pathway | 110 | 190 | 6.14E-27 | 1.46E-25 | ko03015 |
| Human Diseases | Neurodegenerative diseases | Huntington disease | 235 | 566 | 7.86E-27 | 1.71E-25 | ko05016 |
| Metabolism | Amino acid metabolism | Cysteine and methionine metabolism | 80 | 131 | 6.60E-22 | 1.32E-20 | ko00270 |
| Metabolism | Carbohydrate metabolism | Glyoxylate and dicarboxylate metabolism | 77 | 124 | 9.02E-22 | 1.68E-20 | ko00630 |
| Metabolism | Nucleotide metabolism | Pyrimidine metabolism | 72 | 112 | 1.02E-21 | 1.77E-20 | ko00240 |
| Metabolism | Carbohydrate metabolism | Pyruvate metabolism | 83 | 158 | 5.25E-17 | 8.57E-16 | ko00620 |
| Environmental Information Processing | Signal transduction | Phosphatidylinositol signaling system | 82 | 166 | 8.52E-15 | 1.31E-13 | ko04070 |
| Environmental Information Processing | Membrane transport | ABC transporters | 71 | 136 | 1.55E-14 | 2.24E-13 | ko02010 |
| Organismal Systems | Endocrine system | GnRH secretion | 41 | 80 | 1.22E-08 | 1.68E-07 | ko04929 |
| Metabolism | Metabolism of cofactors and vitamins | One carbon pool by folate | 38 | 78 | 2.34E-07 | 3.05E-06 | ko00670 |
| Organismal Systems | Immune system | NOD-like receptor signaling pathway | 44 | 97 | 3.57E-07 | 4.43E-06 | ko04621 |
| Metabolism | Nucleotide metabolism | Purine metabolism | 81 | 222 | 8.01E-07 | 9.50E-06 | ko00230 |
| Human Diseases | Cancers | Acute myeloid leukemia | 34 | 70 | 1.10E-06 | 1.25E-05 | ko05221 |
| Human Diseases | Cancers | Small cell lung cancer | 40 | 92 | 4.49E-06 | 4.89E-05 | ko05222 |
| Organismal Systems | Development | Osteoclast differentiation | 34 | 74 | 5.34E-06 | 5.58E-05 | ko04380 |
| Organismal Systems | Immune system | Toll-like receptor signaling pathway | 34 | 75 | 7.70E-06 | 7.35E-05 | ko04620 |
| Organismal Systems | Immune system | B cell receptor signaling pathway | 34 | 75 | 7.70E-06 | 7.35E-05 | ko04662 |
| Human Diseases | Cancer: overview | PD-L1 expression and PD-1 checkpoint pathway in cancer | 32 | 69 | 7.89E-06 | 7.35E-05 | ko05235 |
| Human Diseases | Cancers | Melanoma | 25 | 49 | 9.72E-06 | 8.75E-05 | ko05218 |
| Environmental Information Processing | Signal transduction | TNF signaling pathway | 34 | 76 | 1.10E-05 | 9.55E-05 | ko04668 |
| Organismal Systems | Endocrine system | Parathyroid hormone synthesis, secretion and action | 36 | 84 | 1.97E-05 | 1.66E-04 | ko04928 |
| Human Diseases | Endocrine and metabolic diseases | Insulin resistance | 45 | 115 | 3.20E-05 | 2.59E-04 | ko04931 |
| Human Diseases | Cancers | Pancreatic cancer | 32 | 73 | 3.28E-05 | 2.59E-04 | ko05212 |
| Organismal Systems | Immune system | Fc epsilon RI signaling pathway | 28 | 61 | 3.66E-05 | 2.81E-04 | ko04664 |
| Environmental Information Processing | Signal transduction | VEGF signaling pathway | 25 | 53 | 5.41E-05 | 3.92E-04 | ko04370 |
| Organismal Systems | Excretory system | Aldosterone-regulated sodium reabsorption | 25 | 53 | 5.41E-05 | 3.92E-04 | ko04960 |
| Human Diseases | Infectious diseases | Chagas disease (American trypanosomiasis) | 32 | 75 | 6.27E-05 | 4.42E-04 | ko05142 |
| Environmental Information Processing | Signal transduction | Jak-STAT signaling pathway | 30 | 69 | 6.92E-05 | 4.71E-04 | ko04630 |
| Organismal Systems | Immune system | T cell receptor signaling pathway | 35 | 85 | 7.03E-05 | 4.71E-04 | ko04660 |
| Human Diseases | Substance dependence | Morphine addiction | 35 | 87 | 0.000123412 | 8.05E-04 | ko05032 |
| Human Diseases | Cancers | Non-small cell lung cancer | 27 | 64 | 0.000288626 | 1.84E-03 | ko05223 |
| Organismal Systems | Nervous system | GABAergic synapse | 34 | 88 | 0.000380712 | 2.26E-03 | ko04727 |
| Human Diseases | Infectious diseases | Measles | 34 | 88 | 0.000380712 | 2.26E-03 | ko05162 |
| Human Diseases | Cancers | Chronic myeloid leukemia | 34 | 88 | 0.000380712 | 2.26E-03 | ko05220 |
| Organismal Systems | Immune system | C-type lectin receptor signaling pathway | 32 | 82 | 0.00045493 | 2.64E-03 | ko04625 |
| Human Diseases | Immune diseases | Asthma | 5 | 5 | 0.000553659 | 3.11E-03 | ko05310 |
| Organismal Systems | Immune system | Natural killer cell mediated cytotoxicity | 26 | 63 | 0.000560662 | 3.11E-03 | ko04650 |
| Human Diseases | Infectious diseases | Kaposi sarcoma-associated herpesvirus infection | 46 | 134 | 0.000906732 | 4.93E-03 | ko05167 |
| Human Diseases | Cancers | Prostate cancer | 33 | 89 | 0.001075551 | 5.73E-03 | ko05215 |
| Human Diseases | Substance dependence | Nicotine addiction | 18 | 40 | 0.001189358 | 6.21E-03 | ko05033 |
