# Supplementary material for: Genomic features of the polyphagous cotton leafworm Spodoptera littoralis
Source: BMC Genomics. 2022 May 7;23:353. doi: 10.1186/s12864-022-08582-w (PMC9080191; doi:10.1186/s12864-022-08582-w)
Supplement: Supplementary file 12 — Additional file 12. [file 12864_2022_8582_MOESM12_ESM.pdf]

(a)

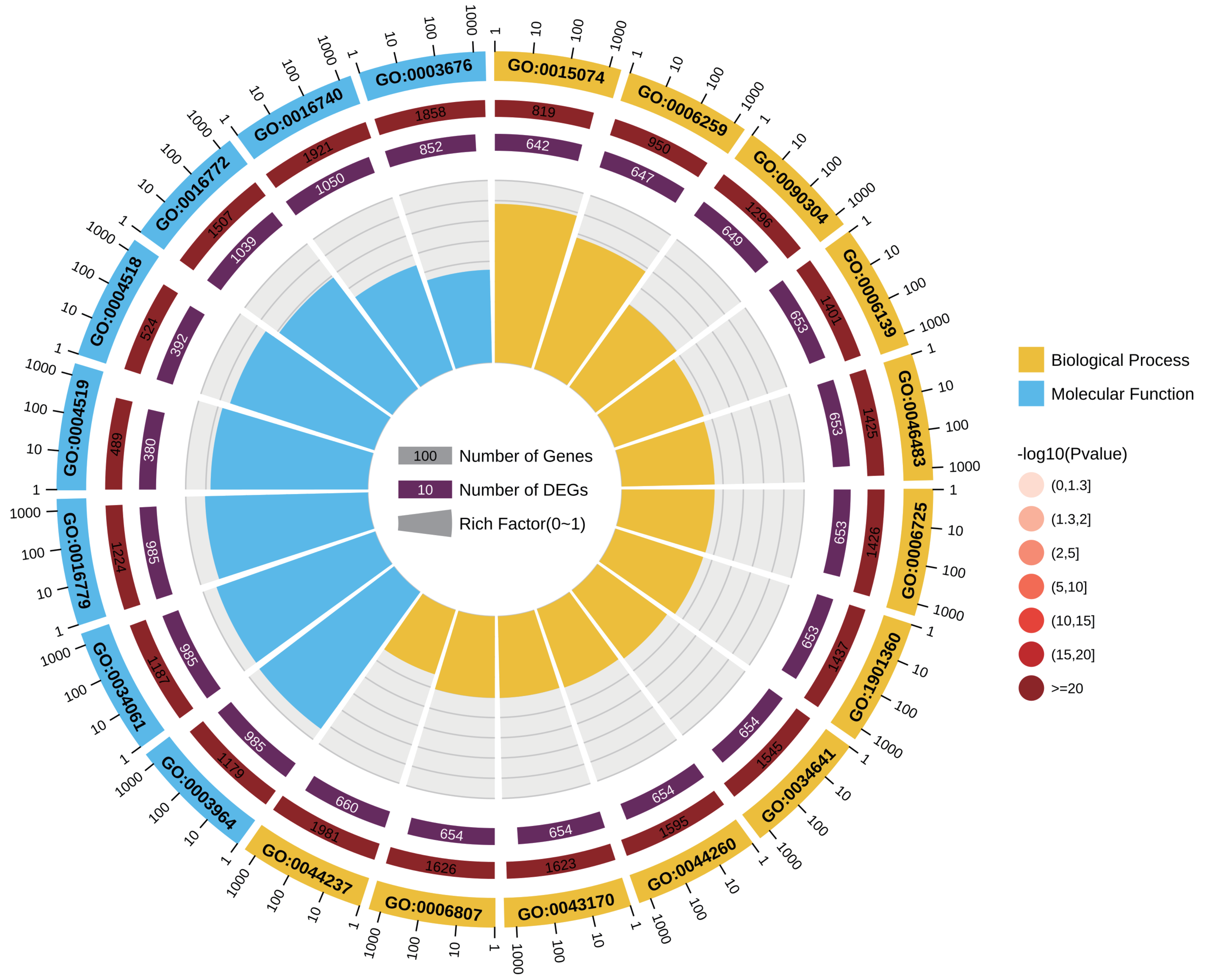

(b)

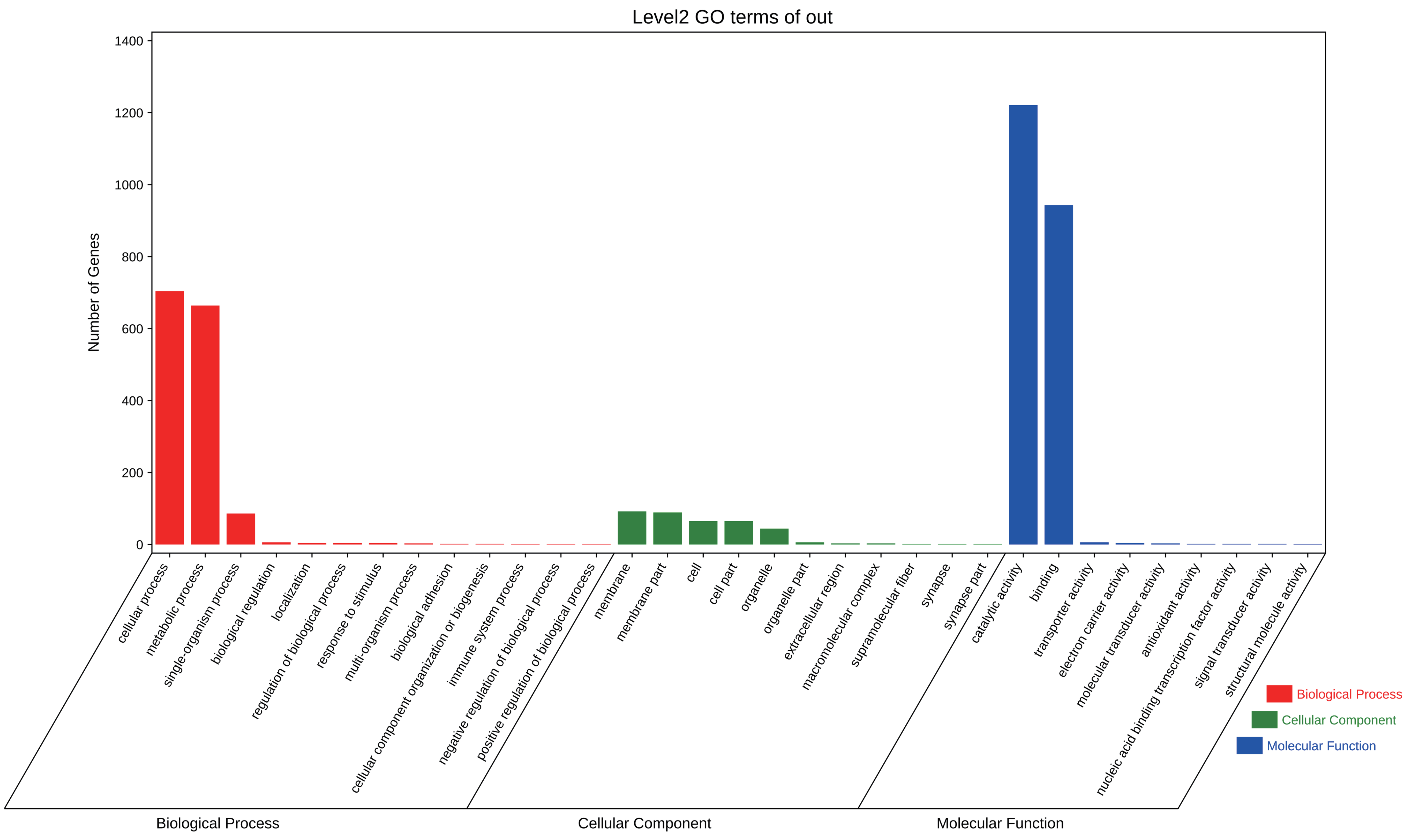

Additional file 12: Fig. S4. GO enrichment of rapidly expanded gene families in *S. littoralis*. (a) Circle diagram of GO enrichment analysis of rapidly expanded gene families (top 20). (b) The gene number of expanded gene families accounted for the number of each GO trem (molecular function, cellular component, biological process) in thelevel2 classification.
