# Supplementary material for: Genomic features of the polyphagous cotton leafworm Spodoptera littoralis
Source: BMC Genomics. 2022 May 7;23:353. doi: 10.1186/s12864-022-08582-w (PMC9080191; doi:10.1186/s12864-022-08582-w)
Supplement: Supplementary file 13 — Additional file 13. [file 12864_2022_8582_MOESM13_ESM.pdf]

(a)

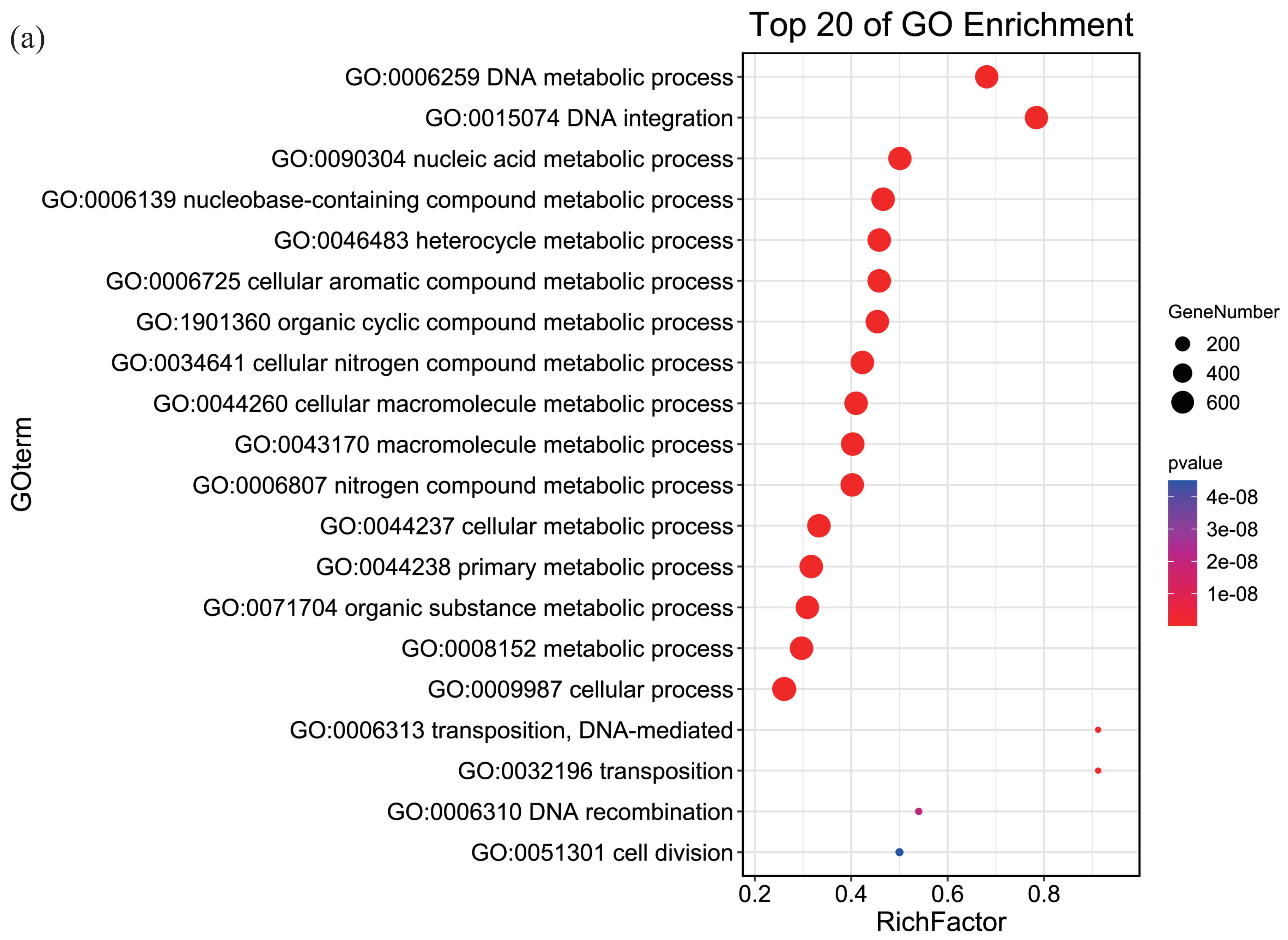

(b)

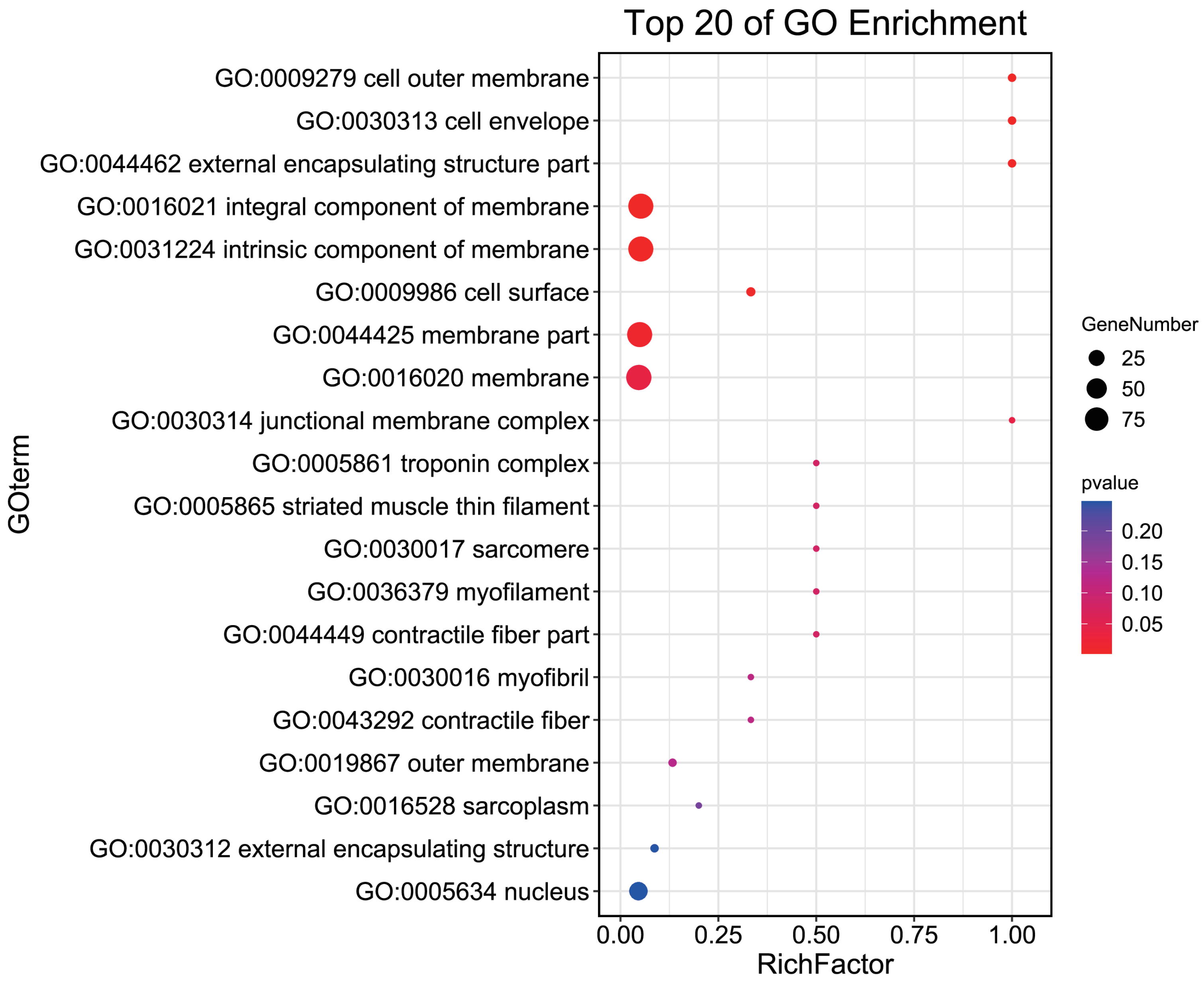

Additional file 13: Fig. S5. Top 20 of GO enrichment on *S. littoralis* rapidly expanded gene families. (a) Biological process category. (b) Cellular component category.
