# Supplementary material for: Genomic features of the polyphagous cotton leafworm Spodoptera littoralis
Source: BMC Genomics. 2022 May 7;23:353. doi: 10.1186/s12864-022-08582-w (PMC9080191; doi:10.1186/s12864-022-08582-w)
Supplement: Supplementary file 14 — Additional file 14. [file 12864_2022_8582_MOESM14_ESM.docx]

Additional file 14: Table S9. GO enrichment analysis on *S. littoralis* rapidly expanded gene families, Biological Process category (padj <0.05)

| GO ID | Description | out (714) | All (3259) | pvalue | p.adjust |
| --- | --- | --- | --- | --- | --- |
| GO:0006259 | DNA metabolic process | 647 | 950 | 0 | 0 |
| GO:0015074 | DNA integration | 642 | 819 | 0 | 0 |
| GO:0090304 | nucleic acid metabolic process | 649 | 1296 | 6.30E-232 | 5.23E-230 |
| GO:0006139 | nucleobase-containing compound metabolic process | 653 | 1401 | 4.07E-209 | 2.54E-207 |
| GO:0046483 | heterocycle metabolic process | 653 | 1425 | 5.27E-203 | 2.62E-201 |
| GO:0006725 | cellular aromatic compound metabolic process | 653 | 1426 | 9.40E-203 | 3.90E-201 |
| GO:1901360 | organic cyclic compound metabolic process | 653 | 1437 | 5.25E-200 | 1.87E-198 |
| GO:0034641 | cellular nitrogen compound metabolic process | 654 | 1545 | 6.50E-176 | 2.02E-174 |
| GO:0044260 | cellular macromolecule metabolic process | 654 | 1595 | 5.33E-165 | 1.48E-163 |
| GO:0043170 | macromolecule metabolic process | 654 | 1623 | 4.16E-159 | 1.04E-157 |
| GO:0006807 | nitrogen compound metabolic process | 654 | 1626 | 1.74E-158 | 3.95E-157 |
| GO:0044237 | cellular metabolic process | 660 | 1981 | 2.39E-101 | 4.95E-100 |
| GO:0044238 | primary metabolic process | 659 | 2076 | 5.66E-86 | 1.08E-84 |
| GO:0071704 | organic substance metabolic process | 660 | 2139 | 5.48E-78 | 9.75E-77 |
| GO:0008152 | metabolic process | 664 | 2238 | 2.45E-68 | 4.07E-67 |
| GO:0009987 | cellular process | 704 | 2699 | 3.45E-51 | 5.37E-50 |
| GO:0006313 | transposition, DNA-mediated | 31 | 34 | 6.49E-18 | 8.98E-17 |
| GO:0032196 | transposition | 31 | 34 | 6.49E-18 | 8.98E-17 |
| GO:0006310 | DNA recombination | 34 | 63 | 2.00E-08 | 2.62E-07 |
| GO:0051301 | cell division | 38 | 76 | 4.54E-08 | 5.65E-07 |
| GO:0000272 | polysaccharide catabolic process | 4 | 4 | 0.00228876 | 0.021919253 |
| GO:0010383 | cell wall polysaccharide metabolic process | 4 | 4 | 0.00228876 | 0.021919253 |
| GO:0010410 | hemicellulose metabolic process | 4 | 4 | 0.00228876 | 0.021919253 |
| GO:0044036 | cell wall macromolecule metabolic process | 4 | 4 | 0.00228876 | 0.021919253 |
| GO:0045491 | xylan metabolic process | 4 | 4 | 0.00228876 | 0.021919253 |
| GO:0045493 | xylan catabolic process | 4 | 4 | 0.00228876 | 0.021919253 |
