# Supplementary material for: Genomic features of the polyphagous cotton leafworm Spodoptera littoralis
Source: BMC Genomics. 2022 May 7;23:353. doi: 10.1186/s12864-022-08582-w (PMC9080191; doi:10.1186/s12864-022-08582-w)
Supplement: Supplementary file 15 — Additional file 15. [file 12864_2022_8582_MOESM15_ESM.docx]

Additional file 15: Table S10. GO enrichment analysis on *S. littoralis* rapidly expanded gene families, Molecular Function category (padj <0.05)

| GO ID | Description | out (1844) | All (6764) | pvalue | p.adjust |
| --- | --- | --- | --- | --- | --- |
| GO:0016772 | transferase activity, transferring phosphorus-containing groups | 1039 | 1507 | 0 | 0 |
| GO:0016779 | nucleotidyltransferase activity | 985 | 1224 | 0 | 0 |
| GO:0034061 | DNA polymerase activity | 985 | 1187 | 0 | 0 |
| GO:0003964 | RNA-directed DNA polymerase activity | 985 | 1179 | 0 | 0 |
| GO:0016740 | transferase activity | 1050 | 1921 | 3.79E-210 | 1.40E-208 |
| GO:0004519 | endonuclease activity | 380 | 489 | 7.30E-130 | 2.25E-128 |
| GO:0004518 | nuclease activity | 392 | 524 | 2.34E-124 | 6.17E-123 |
| GO:0003676 | nucleic acid binding | 852 | 1858 | 5.78E-94 | 1.34E-92 |
| GO:0016788 | hydrolase activity, acting on ester bonds | 396 | 728 | 7.76E-61 | 1.60E-59 |
| GO:0008270 | zinc ion binding | 331 | 574 | 3.93E-58 | 7.28E-57 |
| GO:0046914 | transition metal ion binding | 335 | 707 | 7.56E-34 | 1.27E-32 |
| GO:0004190 | aspartic-type endopeptidase activity | 113 | 173 | 3.95E-26 | 5.62E-25 |
| GO:0070001 | aspartic-type peptidase activity | 113 | 173 | 3.95E-26 | 5.62E-25 |
| GO:1901363 | heterocyclic compound binding | 912 | 2737 | 2.81E-20 | 3.71E-19 |
| GO:0097159 | organic cyclic compound binding | 912 | 2739 | 3.73E-20 | 4.61E-19 |
| GO:0003824 | catalytic activity | 1221 | 3887 | 1.76E-19 | 2.03E-18 |
| GO:0004523 | RNA-DNA hybrid ribonuclease activity | 51 | 105 | 2.26E-06 | 2.46E-05 |
| GO:0016891 | endoribonuclease activity, producing 5'-phosphomonoesters | 51 | 111 | 1.67E-05 | 0.000171299 |
| GO:0016893 | endonuclease activity, active with either ribo- or deoxyribonucleic acids and producing 5'-phosphomonoesters | 51 | 113 | 3.05E-05 | 0.000296686 |
| GO:0004521 | endoribonuclease activity | 51 | 117 | 9.36E-05 | 0.000866116 |
| GO:0004540 | ribonuclease activity | 51 | 123 | 0.000416878 | 0.003672499 |
