# Supplementary material for: Genomic features of the polyphagous cotton leafworm Spodoptera littoralis
Source: BMC Genomics. 2022 May 7;23:353. doi: 10.1186/s12864-022-08582-w (PMC9080191; doi:10.1186/s12864-022-08582-w)
Supplement: Supplementary file 16 — Additional file 16. [file 12864_2022_8582_MOESM16_ESM.docx]

Additional file 16: Table S11. The gene number of GABAB receptor pathways in different lepidopteran insects

| Items | *Species* | Gene number |
| --- | --- | --- |
| oligophagous | *B. mori* | 2 |
|  | *M. sexta* | 0 |
|  | *P. xylostella* | 0 |
| polyphagous | *S. littoralis* | 13 |
|  | *S. litura* | 7 |
|  | *S. frugiperda* | 6 |
|  | *S. exigua* | 11 |
|  | *H. armigera* | 3 |
|  | *T. ni* | 5 |
