# Supplementary material for: Genomic features of the polyphagous cotton leafworm Spodoptera littoralis
Source: BMC Genomics. 2022 May 7;23:353. doi: 10.1186/s12864-022-08582-w (PMC9080191; doi:10.1186/s12864-022-08582-w)
Supplement: Supplementary file 17 — Additional file 17. [file 12864_2022_8582_MOESM17_ESM.docx]

Additional file 17 Table S12. Gene number of detoxification and chemoreception related gene families

| Gene family | *S. littoralis* | *S. litura* | *S. exigua* | *S. frugiperda* | *B. mori* | *D. plexippus* | *H. armigera* | *M. sexta* |
| --- | --- | --- | --- | --- | --- | --- | --- | --- |
| P450 | 120 | 122 | 84 | 182 | 90 | 76 | 106 | 118 |
| UGT | 46 | 52 | 26 | 48 | 42 | 35 | 46 | 40 |
| GST | 34 | 39 | 36 | 58 | 26 | 24 | 39 | 33 |
| PTP | 14 | 16 | 11 | 21 | 15 | 17 | 16 | 18 |
| EPHX | 3 | 6 | 4 | 7 | 7 | 7 | 9 | 24 |
| PTCHD | 7 | 6 | 6 | 9 | 5 | 7 | 5 | 8 |
| ABC transporter | 53 | 54 | 72 | 73 | 56 | 58 | 55 | 60 |
| CCE | 72 | 107 | 68 | 129 | 91 | 55 | 89 | 108 |
| GR | 256 | 237 | 186 | 231 | 76 | 47 | 197 | 65 |
| OR | 69 | 73 | 54 | 86 | 78 | 64 | 83 | 78 |
| CSP | 21 | 23 | 20 | 25 | 21 | 39 | 23 | 22 |
| OBP | 39 | 42 | 36 | 46 | 35 | 29 | 41 | 44 |
| IR | 55 | 60 | 49 | 68 | 65 | 66 | 60 | 64 |
| SNMP | 12 | 14 | 12 | 18 | 16 | 18 | 16 | 19 |

P450, cytochromeP450; UGT, UDP-glucuronosyl transferase; GST, glutathione S-transferase; PTP, protein tyrosine phosphatase; EPHX, epoxide hydrolase; PTCHD, patched domain containing; ABC transporter, ATP binding cassette transporter; CCE, carboxyl/cholinesterase; GR, gustatory receptors; OR, olfactory receptors; CSP, chemosensory proteins; OBP, odorant-binding proteins; IR, ionotropic receptor; SNMP, sensory neuron membrane proteins.
