# Supplementary material for: Genomic features of the polyphagous cotton leafworm Spodoptera littoralis
Source: BMC Genomics. 2022 May 7;23:353. doi: 10.1186/s12864-022-08582-w (PMC9080191; doi:10.1186/s12864-022-08582-w)
Supplement: Supplementary file 18 — Additional file 18. [file 12864_2022_8582_MOESM18_ESM.pdf]

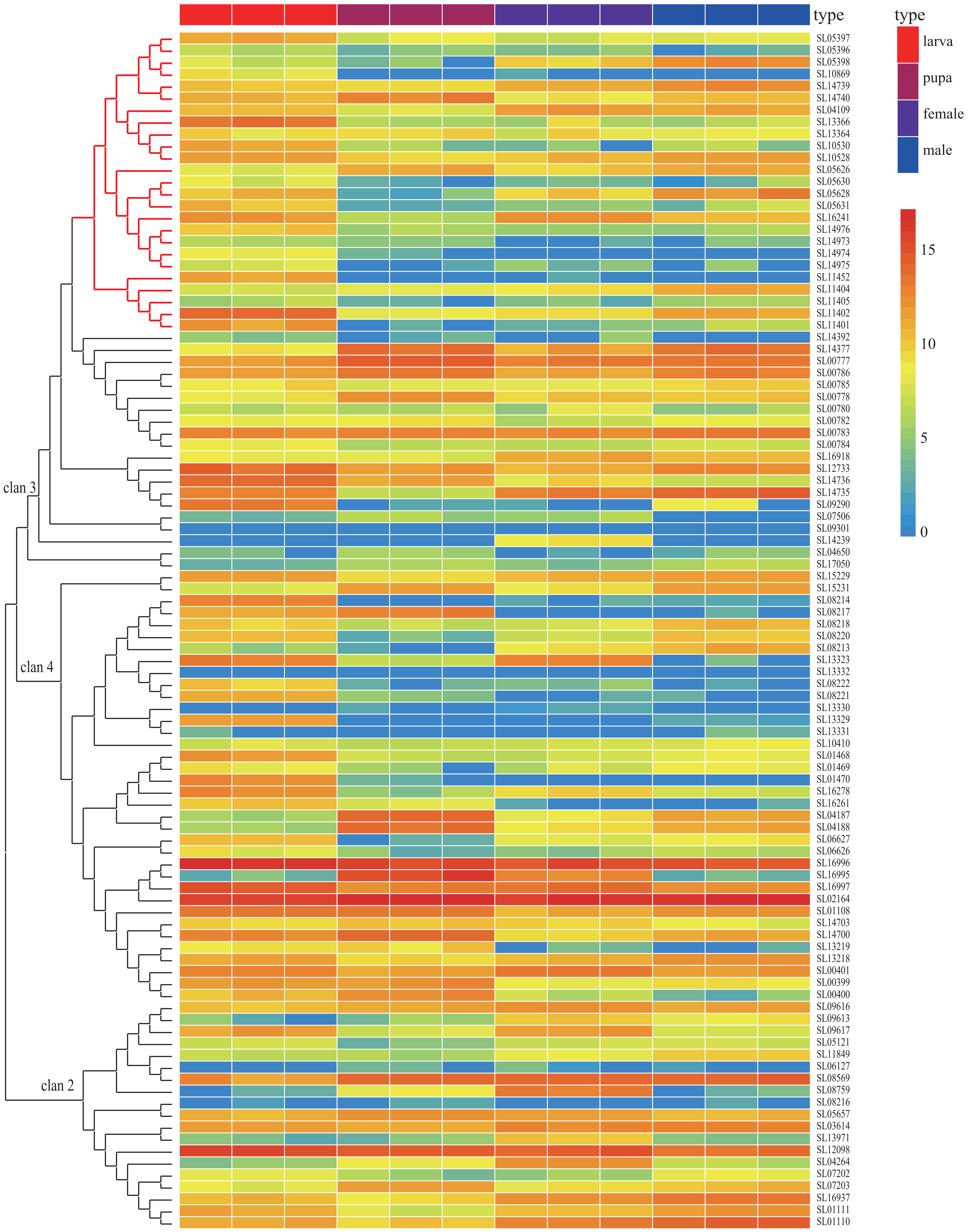

Additional file 18: Fig. S6. Expression levels of P450 genes in different developmental stages of *S. littoralis*. The red branch is the CYP6 subfamily of clan 3.
