# Supplementary material for: Genomic features of the polyphagous cotton leafworm Spodoptera littoralis
Source: BMC Genomics. 2022 May 7;23:353. doi: 10.1186/s12864-022-08582-w (PMC9080191; doi:10.1186/s12864-022-08582-w)
Supplement: Supplementary file 19 — Additional file 19. [file 12864_2022_8582_MOESM19_ESM.pdf]

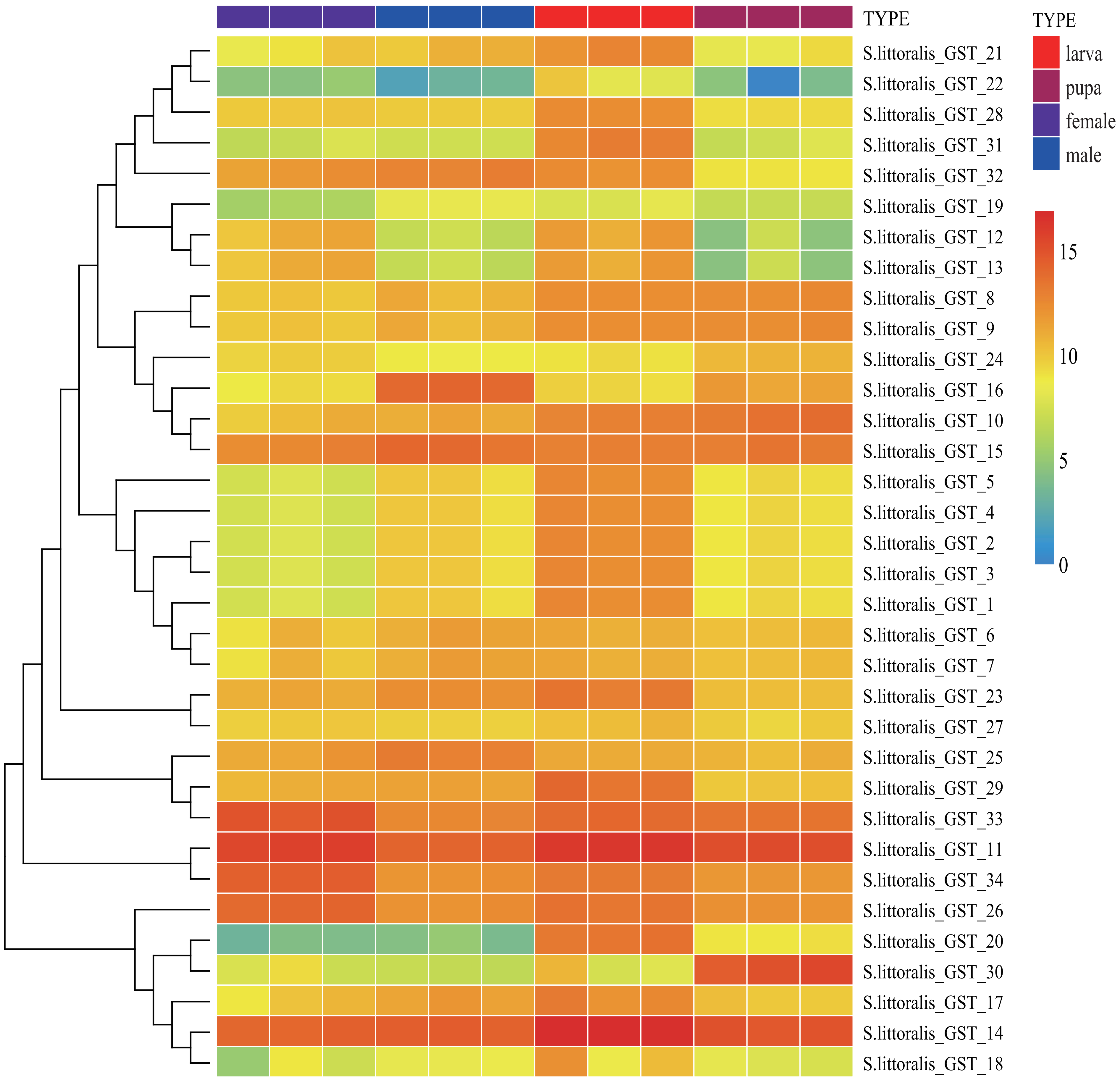

Additional file 19: Fig. S7. Expression levels of GST genes in different developmental stages of *S. littoralis*
