# Supplementary material for: Genomic features of the polyphagous cotton leafworm Spodoptera littoralis
Source: BMC Genomics. 2022 May 7;23:353. doi: 10.1186/s12864-022-08582-w (PMC9080191; doi:10.1186/s12864-022-08582-w)
Supplement: Supplementary file 20 — Additional file 20. [file 12864_2022_8582_MOESM20_ESM.pdf]

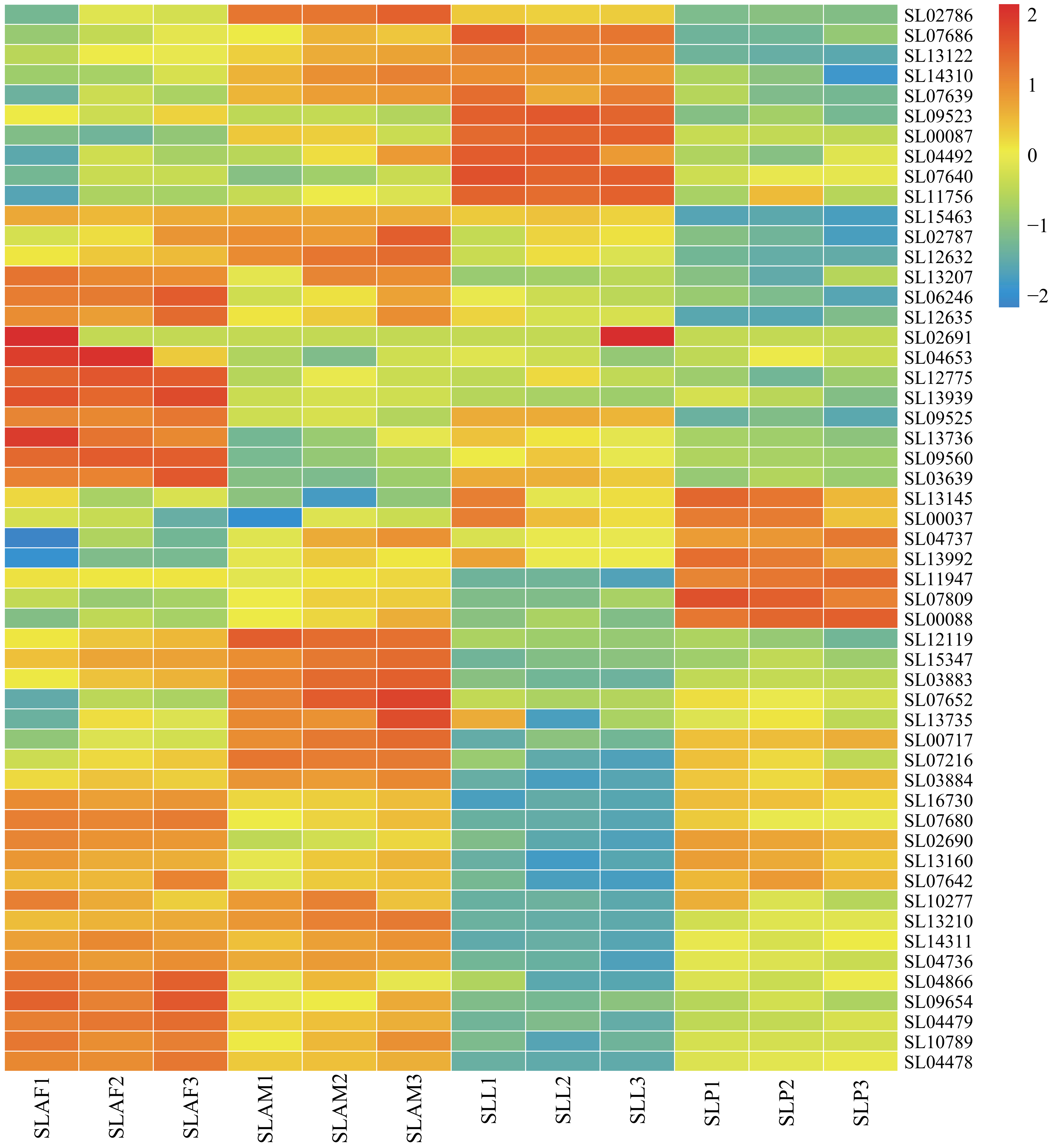

Additional file 20: Fig. S8. Expression levels of ABC transporter genes in different developmental stages of *S. littoralis*. SLAF, female adult; SLAM, male adult; SLL, larva; SLP, pupa.
